# Synthesis of new pyridothienopyrimidinone and pyridothienotriazolopyrimidine derivatives as pim-1 inhibitors

**DOI:** 10.1080/14756366.2017.1389921

**Published:** 2017-11-21

**Authors:** Hala B. El-Nassan, Bassem H. Naguib, Engy A. Beshay

**Affiliations:** a Pharmaceutical Organic Chemistry Department, Faculty of Pharmacy, Cairo University, Cairo, Egypt;; b Pharmaceutical Chemistry Department, Faculty of Pharmacy, The British University in Egypt, Cairo, Egypt;; c National Organization for Drug Control and Research (NODCAR), Cairo, Egypt

**Keywords:** Pyridothienopyrimidine, pyridothienotriazolopyrimidine, cytotoxic activity, pim-1 inhibitors

## Abstract

Three series of 2-arylpyridothieno[3,2-*d*]pyrimidin-4-ones **3a–j**, pyridothienotriazolopyrimidines **6–8** and 4-imino-pyridothieno[3,2-*d*]pyrimidines **9a,b** were prepared to improve the pim-1 inhibitory activity of the previously reported 2-arylpyridothieno[3,2-*d*]pyrimidin-4-ones. All the test compounds showed highly potent pim-1 inhibition with IC_50_ in the range of 0.06–1.76 µM. No significant difference was detected between the pim-1 inhibitory activity of the 4-pyrimidinone and the 4-imino (=NH) or the cyclised triazolopyrimidine derivatives. The most active compounds were tested for their cytotoxic activity on MCF7 and HCT116 and showed potent activity on both the cell lines.

## Introduction

The proviral integration site for Moloney murine leukaemia virus-1 (known as pim-1) is a serine/threonine kinase that controls many cellular functions including cell cycle, cell differentiation, cell survival, apoptosis and drug resistance^1–3^. High levels of pim-1 kinase are associated with many types of cancer such as myeloid leukaemia, breast cancer and prostatic cancer^1–4^. The identification of the role of pim-1 in controlling the growth of cancer stem cells and promotion of multiple drug resistance added more to the importance of developing potent pim-1 inhibitors as anticancer agents that can overcome the drug resistance developed by cancer stem cells^1^^,^^5^.

**Figure 1.**
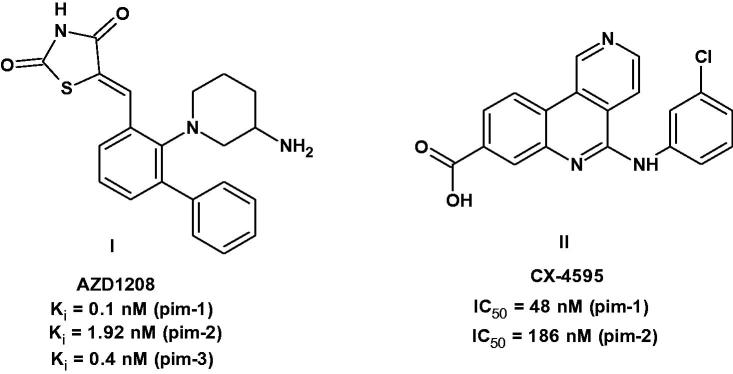
Pim-1 inhibitors under clinical studies.

Within the past 20 years, many pim-1 inhibitors have been identified, developed and are currently under preclinical studies or clinical trials as anticancer agents. Examples include the thiazolidin-2,4-dione derivative AZD1208 (**I**)^1^^,^^2^^,^^6^ and the benzonaphthyridine derivative CX-4595 (**II**)^2^^,^^7^^,^^8^ (Figure 1).

**Figure 2.**
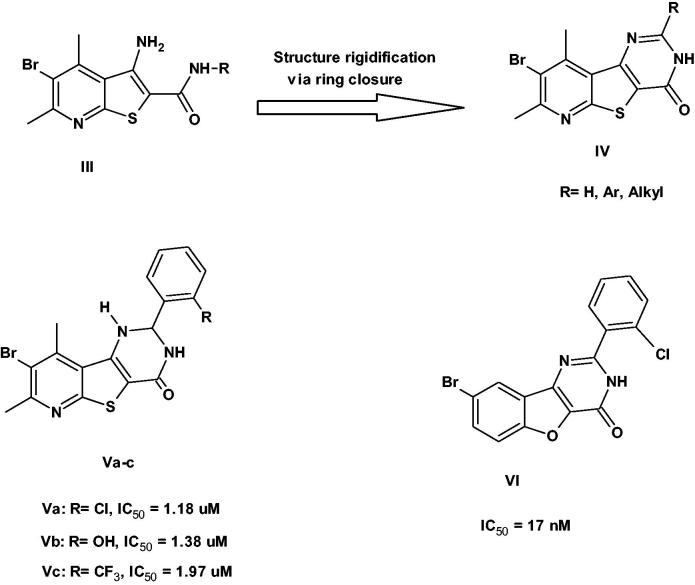
Previous work published on pim-1 inhibitors.

Recently, we had reported the identification of pyridothienopyrimidin-4-one derivatives **IV** as potent pim-1 inhibitors^9^. These derivatives were developed through structure rigidification strategy *via* ring closure of their precursors thieno[2,3-*b*]pyridines **III**^10^ to ensure the presence of the carbonyl group at proper orientation for binding with the enzyme. Indeed, our efforts led to significant improvement in the enzyme inhibitory activity as well as the cytotoxic activity. The most potent inhibitors were the 2-aryl-2,3-dihydro derivatives **Va–c** that exhibited pim-1 inhibition in the range of 1.18–1.97 µM^9^. However, the aryl groups used in that study were all bearing *ortho* substitution to mimic the structure of the previously published benzofuropyrimidinone derivative **VI**^11^ (Figure 2).

**Figure 3.**
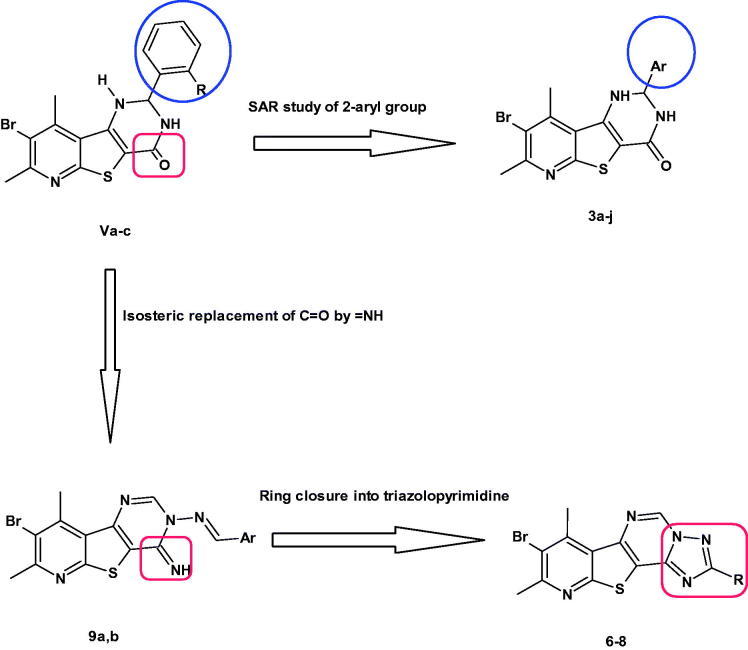
Designing pyridothienopyrimidinones as pim-1 inhibitors.

In continuation to these efforts, we reported herein the SAR study of the effect of substitution on different positions of the aryl group on the pim-1 inhibitory activity. Thus, *para* substitution, disubstitution and trisubstitution on the phenyl ring were all investigated (compounds **3a–j**). Besides, the effects of isosteric replacement of the C=O with = NH to give 4-imino derivatives (**9a,b**) or cyclisation into pyridothienotriazolopyrimidines (**6–8**) on pim-1 inhibition were investigated. It is noteworthy that this is the first published work describing the pim-1 inhibitory activity of pyridothienotriazolopyrimidine derivatives (Figure 3).

All the new compounds were tested for their pim-1 enzyme inhibitory activity and the most active compounds were further tested for their anti-proliferative activity using two different cell lines MCF7 and HCT116.

## Experimental part

### General notes

Stuart SMP20 apparatus was used to determine the melting points and they were uncorrected. The IR spectra were recorded on Shimadzu IR 435 spectrophotometer (Kyoto, Japan) and the values were represented in cm^−1^. The ^1^H NMR and ^13^C NMR spectra were recorded on Bruker 400 and 100 MHz spectrophotometer, respectively. TMS was used as an internal standard and the chemical shifts were recorded in ppm on δ scale. Both IR and NMR spectra were carried out at Faculty of Pharmacy, Cairo University, Cairo, Egypt. The electron impact mass spectra were recorded on Thermo Scientific ISQLT single quadrapole mass spectrometer. Both mass spectra and elemental analyses were carried out at the regional centre for mycology and biotechnology, Al-Azhar University, Cairo, Egypt. All reagents and solvents were purified and dried by standard techniques. 3-Amino-5-bromo-4,6-dimethylthieno[2,3-*b*]pyridine-2-carbonitrile (**1**) and 3-amino-5-bromo-4,6-dimethylthieno[2,3-*b*]pyridine-2-carboxamide **(2)** were prepared according to the published methods^9^^,^^12^.

#### General procedure for the synthesis of 2-aryl-8-bromo-7,9-dimethyl-2,3-dihydropyrido[3′,2′:4,5]thieno[3,2-d]pyrimidin-4(1H)-ones 3a–j

A mixture of 3-amino-5-bromo-4,6-dimethylthieno[2,3-*b*]pyridine-2-carboxamide **(2)** (0.6 g, 0.002 mol) and the appropriate aldehyde (0.002 mol) in glacial acetic acid (10 ml) was heated under reflux for 12 h. The reaction mixture was cooled and the product was filtered, dried and crystallised from acetic acid except **3j** which was crystallised from DMF.

##### 2-(4-(Benzyloxy)phenyl)-8-bromo-7,9-dimethyl-2,3-dihydropyrido[3′,2′:4,5]thieno[3,2-*d*]pyrimidin-4(*1H*)-one (3a)

Yield: 61%; mp: 255–256 °C; IR (cm^−1^): 3421, 3394 (NH), 2924, 2854 (CH-aliphatic), 1654 (C=O); ^1^H NMR (400 MHz, DMSO-d_6_) *δ* ppm 2.70 (s, 3H, CH_3_), 2.85 (s, 3H, CH_3_), 5.08 (s, 2H, OCH_2_), 5.80–5.83 (dd, 1H, CH-2, *J =* 2.96 Hz*, J =* 2.92 Hz), 7.00–7.50 (m, 10H, Ar-H + NH), 8.46 (s, 1H, NH); MS *m/z*: 495 [(M + 2)^+^, 1.77%], 493 [M^+^, 1.89%], 312 [(M + 2-C_6_H_4_OCH_2_C_6_H_5_)^+^, 100%], 310 [(M-C_6_H_4_OCH_2_C_6_H_5_)^+^, 93.00%]; Anal. calcd for C_24_H_20_BrN_3_O_2_S: C, 58.30; H, 4.08; N, 8.50. Found: C, 58.53; H, 4.17; N, 8.79.

##### 8-Bromo-2-(4-bromophenyl)-7,9-dimethyl-2,3-dihydropyrido[3′,2′:4,5]thieno[3,2-*d*]pyrimidin-4(*1H*)-one (3 b)

Yield: 63%; mp: >300 °C; IR (cm^−1^): 3421, 3394 (NH), 2924, 2854 (CH-aliphatic), 1654 (C=O); ^1^H NMR (400 MHz, DMSO-d_6_) *δ* ppm 2.69 (s, 3H, CH_3_), 2.87 (s, 3H, CH_3_), 5.88 (s, 1H, CH-2), 7.18 (d, 1H, NH), 7.46–7.57 (m, 4H, Ar-H), 8.59 (s, 1H, NH); ^13^C NMR (100 MHz, DMSO-d_6_) *δ* ppm 19.9, 26.7 (CH_3_), 65.4 (CH-2), 110.7, 121.4, 121.6, 124.5, 129.1, 131.5, 141.3, 144.2, 144.8, 157.4, 159.5 (Aromatic C), 161.4 (C=O); MS *m/z*: 469 [(M + 4)^+^, 41.60%], 467 [(M + 2)^+^, 100%], 465 [M^+^, 96.83%], 312 [(M + 2-C_6_H_4_Br)^+^, 17.25%], 310 [(M-C_6_H_4_Br)^+^, 14.59%]; Anal. calcd for C_17_H_13_Br_2_N_3_OS: C, 43.71; H, 2.80; N, 8.99. Found: C, 44.05; H, 2.93; N, 9.18.

##### 8-Bromo-2-(4-chlorophenyl)-7,9-dimethyl-2,3-dihydropyrido[3′,2′:4,5]thieno[3,2-*d*]pyrimidin-4(*1H*)-one (3c)

Yield: 68%; mp: >300 °C; IR (cm^−1^): 3394, 3363 (NH), 2924, 2854 (CH-aliphatic), 1666 (C=O); ^1^H NMR (400 MHz, DMSO-d_6_) *δ* ppm 2.69 (s, 3H, CH_3_), 2.87 (s, 3H, CH_3_), 5.90 (s, 1H, CH-2), 7.20–7.53 (m, 4H, Ar-H), 8.30 (s, 1H, NH), 8.60 (s, 1H, NH); Anal. calcd for C_17_H_13_BrClN_3_OS: C, 48.30; H, 3.10; N, 9.94. Found: C, 48.61; H, 3.28; N, 10.11.

##### 8-Bromo-2-(2,4-dihydroxyphenyl)-7,9-dimethyl-2,3-dihydropyrido[3′,2′:4,5]thieno[3,2-*d*]pyrimidin-4(*1H*)-one (3d)

Yield: 68%; mp: 290–291 °C; IR (cm^−1^): 3502–3394 (NH/OH), 2954, 2900 (CH-aliphatic), 1654 (C=O); ^1^H NMR (400 MHz, DMSO-d_6_) *δ* ppm 2.62 (s, 3H, CH_3_), 2.80 (s, 3H, CH_3_), 6.33–6.38 (dd, 1H, CH-2, *J=* 2.12 Hz*, J =* 2.12 Hz), 7.91–8.26 (m, 3H, Ar-H), 8.26 (s, 1H, NH), 10.23 (s, 1H, NH), 12.26 (br s, 2H, OH); Anal. calcd for C_17_H_14_BrN_3_O_3_S: C, 48.58; H, 3.36; N, 10.00. Found: C, 48.75; H, 3.27; N, 10.32.

##### 8-Bromo-2-(3,4-dihydroxyphenyl)-7,9-dimethyl-2,3-dihydropyrido[3′,2′:4,5]thieno[3,2-*d*]pyrimidin-4(*1H*)-one (3e)

Yield: 71%; mp: >300 °C; IR (cm^−1^): 3525 (OH), 3344, 3275 (NH), 2958, 2935 (CH-aliphatic), 1650 (C=O); ^1^H NMR (400 MHz, DMSO-d_6_) *δ* ppm 2.68 (s, 3H, CH_3_), 2.84 (s, 3H, CH_3_), 5.69–5.72 (dd, 1H, CH-2, *J=* 2.88 Hz*, J=* 2.88 Hz), 6.69-6.95 (m, 4H, Ar-H + NH), 8.35 (d, 1H, NH, *J=* 2.92 Hz), 8.90 (s, 1H, OH), 8.95 (s, 1H, OH); ^13^C NMR (100 MHz, DMSO-d_6_) *δ* ppm 19.9, 26.7 (CH_3_), 66.2 (CH-2), 110.5, 114.5, 115.5, 118.1, 121.3, 124.5, 132.3, 144.5, 144.6, 145.3, 145.6, 157.1, 159.4 (Aromatic C), 161.8 (C=O); MS *m/z*: 421 [(M + 2)^+^, 9.58%], 419 [M^+^, 7.50%], 378 [100%]; Anal. calcd for C_17_H_14_BrN_3_O_3_S: C, 48.58; H, 3.36; N, 10.00. Found: C, 48.90; H, 3.49; N, 10.21.

##### 8-Bromo-2-(4-fluorophenyl)-7,9-dimethyl-2,3-dihydropyrido[3′,2′:4,5]thieno[3,2-*d*]pyrimidin-4(*1H*)-one (3f)

Yield: 48%; mp: >300 °C; IR (cm^−1^): 3410, 3170 (NH), 2993, 2954 (CH-aliphatic), 1662 (C=O); ^1^H NMR (400 MHz, DMSO-d_6_) *δ* ppm 2.70 (s, 3H, CH_3_), 2.87 (s, 3H, CH_3_), 5.85 (s, 1H, CH-2), 7.12–7.57 (m, 4H, Ar-H), 8.32 (s, 1H, NH), 8.55 (s, 1H, NH); MS *m/z*: 312 [(M + 2-C_6_H_4_F)^+^, 10.05%], 310 [(M-C_6_H_4_F)^+^, 11.58%]; Anal. calcd for C_17_H_13_BrFN_3_OS: C, 50.26; H, 3.23; N, 10.34. Found: C, 50.41; H, 3.35; N, 10.57.

##### 8-Bromo-2-(4-hydroxy-3-methoxyphenyl)-7,9-dimethyl-2,3-dihydropyrido[3′,2′:4,5]thieno[3,2-*d*]pyrimidin-4(*1H*)-one (3 g)

Yield: 66%; mp: >300 °C; IR (cm^−1^): 3502 (OH), 3394, 3325 (NH), 2997, 2935 (CH-aliphatic), 1647 (C=O); ^1^H NMR (400 MHz, DMSO-d_6_) *δ* ppm 2.69 (s, 3H, CH_3_), 2.84 (s, 3H, CH_3_), 3.76 (s, 3H, OCH_3_), 5.75–5.76 (d, 1H, CH-2), 6.73–7.13 (m, 3H, Ar-H), 7.13 (s, 1H, NH), 8.37 (s, 1H, NH), 9.04 (s, 1H, OH); ^13^C NMR (100 MHz, DMSO-d_6_) *δ* ppm 19.9, 26.7 (CH_3_), 56.0 (OCH_3_), 66.7 (CH-2), 111.1, 111.4, 115.3, 119.6, 121.3, 124.6, 131.8, 144.6, 144.7, 147.0, 147.8, 157.1, 159.5 (Aromatic C), 161.8 (C=O); Anal. calcd for C_18_H_16_BrN_3_O_3_S: C, 49.78; H, 3.71; N, 9.68. Found: C, 50.02; H, 3.89; N, 9.82.

##### 8-Bromo-2-(4-methoxyphenyl)-7,9-dimethyl-2,3-dihydropyrido[3′,2′:4,5]thieno[3,2-*d*]pyrimidin-4(*1H*)-one (3 h)

Yield: 57%; mp: >300 °C; IR (cm^−1^): 3390, 3367 (NH), 2931, 2908 (CH-aliphatic), 1658 (C=O); ^1^H NMR (400 MHz, DMSO-d_6_) *δ* ppm 2.74 (s, 3H, CH_3_), 2.85 (s, 3H, CH_3_), 3.73 (s, 3H, OCH_3_), 5.82 (s, 1H, CH-2), 6.91–7.45 (m, 4H, Ar-H), 8.22 (d, 1H, NH), 8.46 (s, 1H, NH); MS *m/z*: 419 [(M + 2)^+^, 10.28%], 417 [M^+^, 14.49%], 301 [100%]; Anal. calcd for C_18_H_16_BrN_3_O_2_S: C, 51.68; H, 3.86; N, 10.05. Found: C, 51.84; H, 3.98; N, 10.31.

##### 8-Bromo-7,9-dimethyl-2-(3,4,5-trimethoxyphenyl)-2,3-dihydropyrido[3′,2′:4,5]thieno[3,2-*d*]pyrimidin-4(*1H*)-one (3i)

Yield: 59%; mp: >300 °C; IR (cm^−1^): 3236, 3167 (NH), 2989, 2962 (CH-aliphatic), 1651 (C=O); ^1^H NMR (400 MHz, DMSO-d_6_) *δ* ppm 2.71 (s, 3H, CH_3_), 2.87 (s, 3H, CH_3_), 3.70 (br s, 6H, OCH_3_), 3.77 (s, 3H, OCH_3_), 5.81 (s, 1H, CH-2), 6.90–7.00 (m, 3H, Ar-H + NH), 8.48 (s, 1H, NH); Anal. calcd for C_20_H_20_BrN_3_O_4_S: C, 50.22; H, 4.21; N, 8.78. Found: C, 50.49; H, 4.37; N, 8.90.

##### 8-Bromo-7,9-dimethyl-2-(thiophen-3-yl)-2,3-dihydropyrido[3′,2′:4,5]thieno[3,2-*d*]pyrimidin-4(*1H*)-one (3j)

Yield: 54%; mp: 280–281 °C; IR (cm^−1^): 3275, 3190 (NH), 2924, 2854 (CH-aliphatic), 1639 (C=O); ^1^H NMR (400 MHz, DMSO-d_6_) *δ* ppm 2.69 (s, 3H, CH_3_), 2.86 (s, 3H, CH_3_), 5.89–5.91 (dd, 1H, CH-2), 7.06–7.51 (m, 3H, Ar-H), 8.37 (d, 1H, NH), 8.52 (d, 1H, NH); ^13^C NMR (100 MHz, DMSO-d_6_) *δ* ppm 19.9, 26.8 (CH_3_), 63.5 (CH-2), 111.1, 121.3, 123.2, 123.4, 124.8, 127.1, 143.5, 144.4, 144.8, 157.2, 159.4 (Aromatic C), 161.5 (C=O); MS *m/z*: 395 [(M + 2)^+^, 94.00%], 393 [M^+^, 100%]; Anal. calcd for C_15_H_12_BrN_3_OS_2_: C, 45.69; H, 3.07; N, 10.66. Found: C, 45.82; H, 3.22; N, 10.92.

#### Synthesis of ethyl N-(5-bromo-2-cyano-4,6-dimethylthieno[2,3-b]pyridin-3-yl)formimidate (4)

A mixture of 3-amino-5-bromo-4,6-dimethylthieno[2,3-*b*]pyridine-2-carbonitrile **(1)** (0.56 g, 0.002 mol), triethyl orthoformate (6 ml) and acetic anhydride (4 ml) was heated under reflux for 12 h. The reaction mixture was cooled and the product was filtered, dried and crystallised from acetic acid. Yield: 83%; mp: 158–159 °C; IR (cm^−1^): 2989, 2850 (CH aliphatic), 2206 (CN); ^1^H NMR (400 MHz, DMSO-d_6_) *δ* ppm 1.37–1.40 (t, 3H, **CH_3_**CH_2_O, *J =* 6.64 Hz), 2.73 (s, 3H, CH_3_), 2.76 (s, 3H, CH_3_), 4.42–4.44 (q, 2H, CH_3_**CH_2_**O, *J =* 6.76 Hz), 8.35 (s, 1H, =CH); ^13^C NMR (100 MHz, DMSO-d_6_) *δ* ppm 14.5 (**CH_3_**CH_2_), 19.83, 26.9 (ring CH_3_), 64.0 (CH**_3_CH_2_**), 90.9, 105.0, 114.5, 122.9, 145.9, 152.1, 158.1, 159.0, 159.9 (Aromatic C and CN); Anal. calcd for C_13_H_12_BrN_3_OS: C, 46.16; H, 3.58; N, 12.42. Found: C, 45.93; H, 3.72; N, 12.69.

#### Synthesis of 8-bromo-4-imino-7,9-dimethylpyrido[3′,2′:4,5]thieno[3,2-d]pyrimidin-3(4H)-amine (5)

Compound **4** (0.67 g, 0.002 mol) was mixed with hydrazine hydrate (99%, 6 ml) in absolute ethanol (10 ml). The mixture was heated under reflux for 10 h, allowed to cool and the product was filtered, dried and crystallised from acetic acid. Yield: 90%; mp: >300 °C; IR (cm^−1^): 3367, 3329, 3294 (NH/NH_2_), 2951, 2920 (CH aliphatic), 1658 (C = N); ^1^H NMR (400 MHz, DMSO-d_6_) *δ* ppm 2.76 (s, 3H, CH_3_), 3.15 (s, 3H, CH_3_), 4.95 (s, 2H, NH_2_), 8.57 (s, 1H, =CH), 9.18 (s, 1H, =NH); Anal. calcd for C_11_H_10_BrN_5_S: C, 40.75; H, 3.11; N, 21.60. Found: C, 40.91; H, 3.24; N, 21.93.

#### Synthesis of 8-bromo-7,9-dimethylpyrido[3′,2′:4,5]thieno[2,3-e][1,2,4]triazolo[1,5-c]pyrimidine (6)

A mixture of compound **5** (0.64 g, 0.002 mol) and formic acid (3 ml) was heated under reflux for 5 h. The reaction mixture was allowed to cool and poured onto ice-cold water (20 ml), then the product was filtered, dried and crystallised from acetic acid. Yield: 73%; mp: 277–278 °C; IR (cm^−1^): 2985, 2962 (CH aliphatic), 1616 (C = N); ^1^H NMR (400 MHz, DMSO-d_6_) *δ* ppm 2.76 (s, 3H, CH_3_), 3.12 (s, 3H, CH_3_), 8.82 (s, 1H, =CH), 9.94 (s, 1H, =CH); Anal. calcd for C_12_H_8_BrN_5_S: C, 43.13; H, 2.41; N, 20.96. Found: C, 43.50; H, 2.53; N, 21.08.

#### Synthesis of 8-bromo-2,7,9-trimethylpyrido[3′,2′:4,5]thieno[2,3-e][1,2,4]triazolo[1,5-c]pyrimidine (7a) and 8-bromo-7,9-dimethyl-2-(trifluoromethyl)pyrido[3′,2′:4,5]thieno[2,3-e][1,2,4]triazolo[1,5-c]pyrimidine (7 b)

A mixture of compound **5** (0.64 g, 0.002 mol) and acetic anhydride or 2,2,2-trifluoroacetic anhydride (4 ml) was heated under reflux for 5 h and then allowed to cool. The reaction mixture was poured onto ice-cold water (100 ml) and the product was filtered, dried and crystallised from acetic acid.

#### 8-Bromo-2,7,9-trimethylpyrido[3′,2′:4,5]thieno[2,3-e][1,2,4]triazolo[1,5-c]pyrimidine (7a)

Yield: 93%; mp: 218–219 °C; IR (cm^−1^): 2939, 2850 (CH aliphatic), 1612 (C = N); ^1^H NMR (400 MHz, DMSO-d_6_) *δ* ppm 1.91 (s, 3H, CH_3_), 2.80 (s, 3H, CH_3_), 3.19 (s, 3H, CH_3_), 9.18 (s 1H, =CH); Anal. calcd for C_13_H_10_BrN_5_S: C, 44.84; H, 2.89; N, 20.11. Found: C, 45.12; H, 3.01; N, 20.43.

#### 8-Bromo-7,9-dimethyl-2-(trifluoromethyl)pyrido[3′,2′:4,5]thieno[2,3-e][1,2,4]triazolo[1,5-c]pyrimidine (7 b)

Yield: 68%; mp: 199–200 °C; IR (cm^−1^): 2958, 2924 (CH aliphatic), 1612 (C = N); ^1^H NMR (400 MHz, DMSO-d_6_) *δ* ppm 2.67 (s, 3H, CH_3_), 3.01 (s, 3H, CH_3_), 8.69 (s, 1H, =CH); ^13^C NMR (100 MHz, DMSO-d_6_) *δ* ppm 19.1, 26.5 (CH_3_), 117.2, 119.0, 123.2, 123.9, 135.7, 139.7, 141.7, 145.4, 146.5, 148.8, 158.5 (Aromatic C and CF_3_); Anal. calcd for C_13_H_7_BrF_3_N_5_S: C, 38.82; H, 1.75; N, 17.41. Found: C, 39.07; H, 1.89; N, 17.68.

#### Synthesis of ethyl 2-(8-bromo-7,9-dimethylpyrido[3′,2′:4,5]thieno[2,3-e][1,2,4]triazolo[1,5-c]pyrimidin-2-yl)acetate (8)

A mixture of compound **5** (0.64 g, 0.002 mol) and diethyl malonate (5 ml) was heated under reflux for 2 h and then allowed to cool. The product was filtered, dried and crystallised from acetic acid. Yield: 27%; mp: 198–199 °C; IR (cm^−1^): 2978, 2924 (CH aliphatic), 1735 (ester C=O); ^1^H NMR (400 MHz, DMSO-d_6_) *δ* ppm 1.22–1.25 (t, 3H, **CH_3_**CH_2_, *J =* 7.04 Hz), 2.51 (s, 3H, CH_3_), 2.84 (s, 3H, CH_3_), 4.11-4.19 (q, 2H, **CH_2_**CH_3_, *J =* 7.04 Hz), 4.59 (s, 2H, CH_2_COO), 9.40 (s, 1H, =CH); Anal. calcd for C_16_H_14_BrN_5_O_2_S: C, 45.72; H, 3.36; N, 16.66. Found: C, 45.98; H, 3.54; N, 16.93.

#### Synthesis of 8-bromo-N-(substituted benzylidene)-4-imino-7,9-dimethylpyrido[3′,2′:4,5]thieno[3,2-d]pyrimidin-3(4H)-amines 9a,b

A mixture of compound **5** (0.64 g, 0.002 mol) and 4-bromobenzaldehyde or 2-(trifluoromethyl)benzaldehyde (0.002 mol) in glacial acetic acid (10 ml) was heated under reflux for 7 h and then allowed to cool. The product was filtered, dried and crystallised from acetic acid.

#### 8-Bromo-N-(4-bromobenzylidene)-4-imino-7,9-dimethylpyrido[3′,2′:4,5]thieno[3,2-d]pyrimidin-3(4H)-amine (9a)

Yield: 90%; mp: >300 °C; IR (cm^−1^): 3197 (NH), 2920, 2850 (CH aliphatic); ^1^H NMR (400 MHz, DMSO-d_6_) *δ* ppm 2.81 (s, 3H, CH_3_), 3.32 (s, 3H, CH_3_), 7.78–8.22 (m, 5H, Ar-H + NH), 8.32 (s, 1H, CH-2), 8.80 (s, 1H, N=CH); Anal. calcd for C_18_H_13_Br_2_N_5_S: C, 44.01; H, 2.67; N, 14.26. Found: C, 43.89; H, 2.80; N, 14.38.

#### 8-Bromo-4-imino-7,9-dimethyl-N-(2-(trifluoromethyl)benzylidene)pyrido[3′,2′:4,5]thieno[3,2-d]pyrimidin-3(4H)-amine (9 b)

Yield: 78%; mp: >300 °C; IR (cm^−1^): 3178 (NH), 2970, 2819 (CH aliphatic); ^1^H NMR (400 MHz, DMSO-d_6_) *δ* ppm 2.67 (s, 3H, CH_3_), 3.05 (s, 3H, CH_3_), 7.61–7.89 (m, 5H, Ar-H + NH), 8.51 (s, 1H, CH-2), 8.68 (s, 1H, N=CH); Anal. calcd for C_19_H_13_BrF_3_N_5_S: C, 47.51; H, 2.73; N, 14.58. Found: C, 47.82; H, 2.86; N, 14.74.

#### Pim-1 kinase inhibitory activity

**Figure 4.**
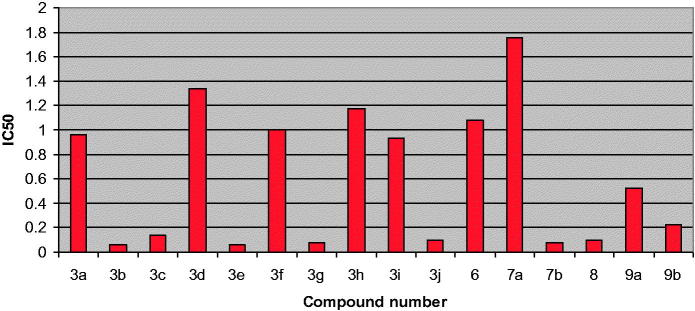
IC_50_ of the synthesised compounds in µM on pim-1 kinase.

The kinase inhibitory activity of the synthesised compounds was determined using Human proto-oncogene serine/threonine-protein kinase pim-1 (PIM1) ELISA Kit (catalog #MBS7573). All the compounds were tested for their inhibitory activity against pim-1 kinase at 0.2, 1, 5 and 25 µM using Staurosporine as a reference standard. The results were displayed in terms of percent inhibition and IC_50_. Table 1 and Figure 4 show the obtained results.

**Table 1.** Results of pim-1 kinase inhibition achieved by the test compounds.


Compound number	R or Ar	% Inhibition at 25µM	IC_50_ (µM)
3a	4-C_6_H_5_CH_2_OC_6_H_4_	83	0.96
3b	4-BrC_6_H_4_	96	0.06
3c	4-ClC_6_H_4_	92	0.14
3d	2,4-(OH)_2_C_6_H_3_	81	1.34
3e	3,4-(OH)_2_C_6_H_3_	93	0.06
3f	4-FC_6_H_4_	89	1.00
3g	3-OCH_3_-4-OHC_6_H_3_	92	0.08
3h	4-OCH_3_C_6_H_4_	84	1.17
3i	3,4,5-(OCH_3_)_3_C_6_H_2_	81	0.93
3j	3-Thienyl	95	0.10
6	H	81	1.08
7a	CH_3_	76	1.76
7b	CF_3_	93	0.08
8	CH_2_COOC_2_H_5_	95	0.10
9a	4-BrC_6_H_4_	88	0.52
9b	2-CF_3_C_6_H_4_	89	0.22
Staurosporine	–	94	Not determined

#### *In vitro* cytotoxic activity

#### Cell culture protocol

Cell line cells were obtained from American Type Culture Collection (ATCC, Manassas, VA). The cell lines used in this study were human breast adenocarcinoma (MCF7) and human colon adenocarcinoma (HCT116). The cells were cultured using DMEM (Invitrogen/Life Technologies) supplemented with 10% FBS (Hyclone), 10 µg/mL insulin (Sigma) and 1% penicillin-streptomycin. All of the other chemicals and reagents were from Sigma or Invitrogen.

Plate cells (cells density 1.2–1.8 × 10,000 cells/well) in a volume of 100 µL complete growth medium and 100 µL of the test compound per well were prepared in a 96-well plate for 24 h before the MTT assay. The culture medium was removed to a centrifuge tube. The cell layer was rinsed with 0.25% (w/v) Trypsin 0.53 mM EDTA solution to remove all traces of serum which contains Trypsin inhibitor. Then, 2–3 ml of Trypsin EDTA solution was added and the cells were observed under an inverted microscope until the cell layer is dispersed (usually within 5–15 min.). A volume of 6 to 8 ml of complete growth medium was added and the cells were aspirated by gently pipetting. The cell suspension was transferred to the centrifuge tube with the culture medium and centrifuged at approximately 125 ×*g* for 5 to 10 min. The supernatant was discarded and the cells were suspended in fresh growth medium then incubated at 37 °C for 24 h. After treatment of the cells with the serial concentrations of the test compounds or the control Staurosporine in DMSO (at concentrations of 200, 100, 10, 1 and 0.1 µM), incubation was carried out for 48 h at 37 °C, then the plates were examined under the inverted microscope and proceeded for the MTT assay.

#### Cytotoxicity assay by 3-[4,5-dimethylthiazol-2-yl]-2,5-diphenyltetrazolium bromide (MTT)

**Figure 5.**
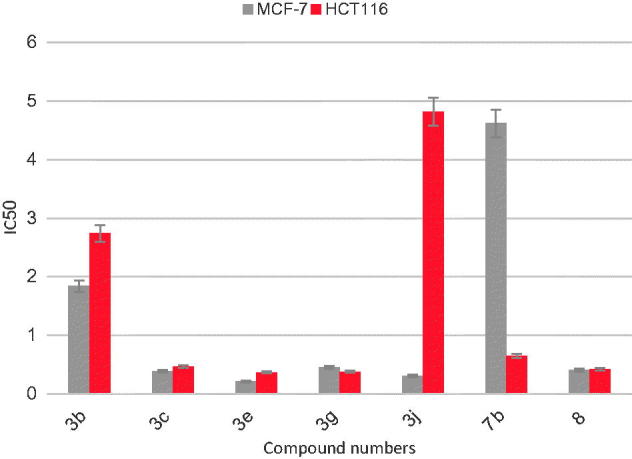
IC_50_ in µM of compounds **3b, 3c, 3e, 3g, 3j, 7b** and **8** on MCF7 and HCT116 cell lines.

The MTT method of monitoring *in vitro* cytotoxicity is well suited for use with multiwell plates. For best results, cells in the log phase of growth should be employed and final cell number should not exceed 10^6^ cells/cm^2^. Each test should include a blank containing complete medium without cells. Cell cultures were removed from the incubator into laminar flow hood. Each vial of MTT [M-5655] was reconstituted to be used with 3 ml of medium or balanced salt solution without phenol red and serum and the reconstituted MTT was added in an amount equal to 10% of the culture medium volume. Then the cultures were returned to the incubator for 2–4 h depending on the cell type and the maximum cell density. After the incubation period, the cultures were removed from incubator and the resulting formazan crystals were dissolved by adding an amount of MTT solvent (solubilisation solution) [M-8910] equal to the original culture medium volume. Gentle mixing in a gyratory shaker enhanced dissolution. Occasionally pipetting up and down [trituration] may be required to completely dissolve the MTT formazan crystals. The absorbance was measured at a wavelength of 450 nm using Bioline ELIZA reader. The background absorbance of the multiwell plates was measured at 690 nm and subtracted from the 450 nm measurement. The results are shown in Table 2 and graphically represented in Figure 5.

**Table 2.** Results of log P and *in vitro* cytotoxic screening of compounds **3b, 3c, 3e, 3g, 3j, 7b** and **8** on two cell lines.

	IC_50_ μM^a^		
Compound number	MCF-7	HCT116	log P
3b	1.84 ± 0.12	2.74 ± 0.15	5.02
3c	0.39 ± 0.07	0.47 ± 0.03	4.88
3e	0.22 ± 0.04	0.37 ± 0.02	3.24
3g	0.46 ± 0.09	0.38 ± 0.05	3.55
3j	0.31 ± 0.02	4.82 ± 0.21	3.79
7b	4.62 ± 0.14	0.65 ± 0.04	3.89
8	0.41 ± 0.03	0.42 ± 0.01	3.38

a The values given are means of three experiments.

## Results and discussion

### Chemistry

**Scheme 1.**
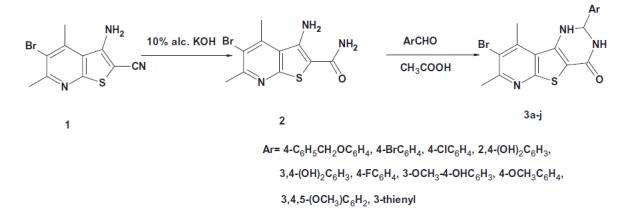
Synthesis of the target compounds **3a–j**.

**Scheme 2.**
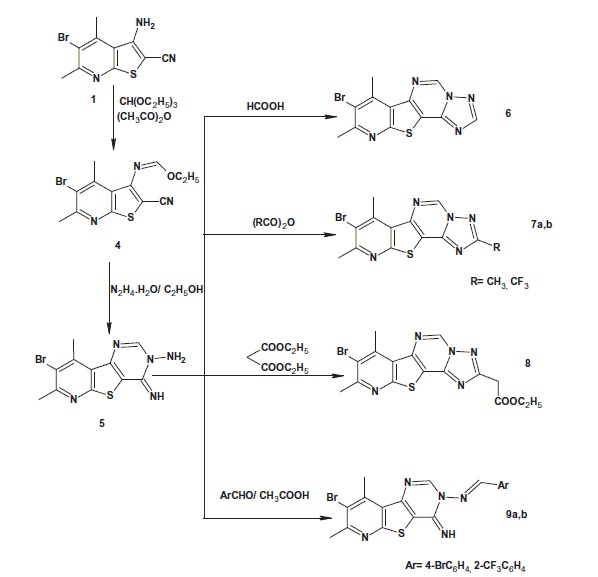
Synthesis of the target compounds **6–9**.

Schemes 1 and 2 outline the synthesis of the target compounds. In the current work, 3-amino-5-bromo-4,6-dimethylthieno[2,3-b]pyridine-2-carboxamide **(2)** was prepared by alkaline hydrolysis of the 3-aminothieno[2,3-*b*]pyridine-2-carbonitrile **1** as reported by Naguib et al^9^. Compound **2** was then reacted with different aldehydes in glacial acetic acid to afford 2-aryl-8-bromo-2,3-dihydropyrido[3′,2′:4,5]thieno[3,2-*d*]pyrimidin-4(*1H*)-ones **3a–j.** The IR spectra of compounds **3a–j** showed two NH bands at 3502–3167 cm^−1^ together with C=O band at 1666–1639 cm^−1^. Their ^1^H NMR spectra revealed the presence of a signal at δ 5.69–6.38 ppm corresponding to CH-2 proton. Besides, two signals appeared at δ 6.90–8.37 ppm and δ 8.35–10.23 ppm corresponding to two NH protons together with the aromatic protons signals at δ 6.69–8.26 ppm. The ^13^C NMR spectra of compounds **3b**, **3e**, **3g and3j** showed a signal at δ 63.5–66.7 ppm corresponding to CH-2 carbon and a signal at δ 161.4–161.8 ppm corresponding to C=O carbon. Finally, the mass spectra of compounds **3a, 3b, 3e, 3h** and **3j** showed their corresponding molecular ion peaks.

On the other hand, reacting 3-aminothieno[2,3-*b*]pyridine-2-carbonitrile **1** with triethyl orthoformate and acetic anhydride afforded the ethyl formimidate derivative **4**. The formation of the latter compound was confirmed by IR spectroscopy that revealed the disappearance of the characteristic NH_2_ forked bands. Its ^1^H NMR spectrum showed triplet and quartet signals at δ 1.37–1.40 ppm and δ 4.42–4.44 ppm corresponding to CH_3_CH_2_ protons, respectively. Moreover, a singlet signal appeared at δ 8.35 ppm corresponding to N=CH proton. The ^13^C NMR spectrum of compound **4** revealed the presence of CH_3_CH_2_ carbons at δ 14.5 and 64.0 ppm, respectively.

Compound **4** was allowed to react with hydrazine hydrate to give 8-bromo-4-imino-7,9-dimethylpyrido[3′,2′:4,5]thieno[3,2-*d*]pyrimidin-3(*4H*)-amine (**5**). The IR spectrum of compound **5** showed three bands at 3367, 3329, 3294 cm^−1^ corresponding to NH/NH_2_ groups. The ^1^H NMR spectrum of compound **5** showed two singlet signals at δ 4.95 ppm and δ 9.18 ppm corresponding to NH_2_ and = NH protons, respectively, in addition to a singlet signal at δ 8.57 ppm corresponding to CH-2 proton.

Reacting compound **5** with formic acid or different anhydrides afforded pyrido[3′,2′:4,5]thieno[2,3-*e*][1,2,4]triazolo[1,5-*c*]pyrimidine derivatives **6** and **7a,b**, respectively. Their IR and ^1^H NMR spectra revealed the absence of NH/NH_2_ groups.

The acetate derivative **8** was obtained via reacting compound **5** with diethyl malonate. The IR spectrum of compound **8** indicated the presence of the ester carbonyl band at 1735 cm^−1^. The ^1^H NMR spectrum revealed the appearance of a singlet signal at δ 4.59 ppm assigned to the methylene protons in addition to triplet and quartet signals at δ 1.22–1.25 ppm and δ 4.11–4.19 ppm corresponding to the ethyl protons.

Finally, the reaction of compound **5** with different aldehydes in glacial acetic acid furnished *N*-(substituted benzylidene)-4-imino-pyridothienopyrimidin-3-amines **9a,b**. Their IR spectra indicated the disappearance of NH_2_ band, while their ^1^H NMR spectra revealed the presence of singlet signal of N=CH proton as well as the introduced aromatic protons.

### Pim-1 kinase inhibitory activity

All the compounds were tested for their ability to inhibit pim-1 kinase at 0.2, 1, 5 and 25 µM using Staurosporine as a reference compound and the results in terms of percentage inhibition and IC_50_ were displayed in Table 1 and represented graphically in Figure 4.

The results indicated that all the test compounds exhibited highly potent pim-1 inhibitory activity in the range of 76–96% at 25 µM and IC_50_ in the range of 0.06–1.76 µM. The most potent compounds were **3b** (96% inhibition, IC_50_= 0.06 µM), **3c** (92% inhibition, IC_50_= 0.14 µM), **3e** (93% inhibition, IC_50_= 0.06 µM), **3g** (92% inhibition, IC_50_= 0.08 µM), **3j** (95% inhibition, IC_50_= 0.10 µM), **7b** (93% inhibition, IC_50_= 0.08 µM) and **8** (95% inhibition, IC_50_= 0.10 µM).

SAR study of the 2-aryl-8-bromo-7,9-dimethyl-2,3-dihydropyrido[3′,2′:4,5]thieno[3,2-*d*]pyrimidin-4(*1H*)-ones **3a–j** as pim-1 inhibitors indicated the following points:

- Regarding the *para*-substituted derivatives **3a–c, f** and **h**, the best results were obtained with halogens especially **3b** (4-bromophenyl) with IC_50_ = 0.06 µM and **3c** (4-chlorophenyl) with IC_50_ = 0.14 µM. Increasing the size of the substituents from **3h** (4-methoxyphenyl) to **3a** (4-benzyloxyphenyl) improved the inhibitory activity slightly.
- Regarding the disubstituted derivatives **3d, e, g**, better results were obtained with 3,4-disubstituted than with 2,4-disubstituted analogues. Both **3g** (3-OCH_3_-4-OH derivative) and **3e** (3,4-dihydroxy derivative) gave highly potent pim-1 inhibitory activity with IC_50_ of 0.08 and 0.06 µM, respectively.
- Increasing the number of substitution in **3i** (3,4,5-trimethoxyphenyl) was accompanied by reduction in the inhibitory activity (IC_50_=0.93 µM).
- The presence of the heterocyclic ring (3-thienyl) in **3j** gave highly potent pim-1 inhibitor as well (IC_50_=0.10 µM).

Upon designing the target pyridothienopyrimidinones, it was assumed that the substituent at position 2 of the ring occupied a solvent exposed area in pim-1 kinase active site and thus can accommodate various groups. Introduction of H-bonding and hydrophilic groups (like OH and methoxy groups) on the aryl ring especially at *meta* and *para* positions seemed to make extra H-bonding to the enzyme active site which might account for the higher kinase inhibitory activity exerted by compounds **3e** and **3g**.

On the other hand, SAR study of the pyridothienotriazolopyrimidines **6–8** indicated the following remarks:

- The unsubstituted triazole derivative **6** showed potent pim-1 inhibition with IC_50_= 1.08 µM.
- Substitution with methyl group in compound **7a** reduced the inhibitory activity. However, substitution with CF_3_ group in compound **7b** or C_2_H_5_OCOCH_2_ moiety in compound **8** improved the inhibitory activity significantly.

Finally, the 4-imino derivative **9a, b** showed also potent pim-1 inhibitory activity with IC_50_= 0.52 and 0.22 µM, respectively. It is noteworthy that no significant difference was detected between the pim-1 inhibitory activity of the 4-pyrimidinone and the 4-imino (=NH) or the cyclised triazolopyrimidine derivatives.

### *In vitro* cytotoxic activity

The most active pim-1 inhibitors in this work (compounds **3b**, **3c**, **3e**, **3g**, **3j**, **7b** and **8**) were tested for their cytotoxic activity against MCF7 and HCT116 cell lines using MTT method^13^^,^^14^. The results in terms of IC_50_ in µM are given in Table 2 and represented graphically in Figure 5.

All the compounds showed potent cytotoxic activity on both cell lines. The cytotoxic activity on MCF7 cell line ranged between 0.22 and 1.84 µM. Five compounds (**3c**, **3e**, **3g**, **3j** and **8**) exerted potent cytotoxic activity with IC_50_ values below 0.5 µM. Whilst, the cytotoxic activity on HCT116 ranged between 0.37 and 4.82 µM. Here five compounds (**3c**, **3e**, **3g**, **7b** and **8**) showed potent cytotoxic activity with IC_50_ values below 1 µM.

Compound **3e** [the 2-(3,4-dihydroxyphenyl) derivative] displayed the most potent cytotoxic effect on both cell lines with IC_50_ values of 0.22 and 0.37 µM, respectively. These results were consistent with its high kinase inhibitory activity (IC_50_= 0.06 µM). While compound **3b** showed the least potent cytotoxic activity on both cell lines with IC_50_ values of 1.84 and 2.74 µM, respectively.

Notably, compounds **3b** and **3e** exhibited the same pim-1 inhibitory activity with IC_50_ of 0.06 µM. The dramatic variation in their cytotoxic activity might be explained by examining their calculated log *p* values^15^ (Table 2). The calculated log P of compound **3b** was 5.02, while that of **3e** was 3.24.

It is noteworthy that significant improvement was observed in the cytotoxic activity of the tested compounds compared to their precursors **Va–c**. The latter cytotoxicity range was between 30 and 180 µM on the same cell lines^9^.

## Conclusions

In this work, series of 2-arylpyridothieno[3,2-*d*]pyrimidin-4-ones **3a–j**, pyridothienotriazolopyrimidine **6–8** and 4-imino-pyridothieno[3,2-*d*]pyrimidines **9a,b** were prepared to improve the pim-1 inhibitory activity of the previously reported 2-arylpyridothieno[3,2-*d*]pyrimidin-4-ones. The lead optimisation strategies used were: isosteric replacement of C=O by =NH and ring closure of the imino derivatives into triazolo ring. Besides, a brief SAR study of the substitution on the 2-aryl moiety was done. All the test compounds showed highly potent pim-1 inhibition with IC_50_ in the range of 0.06–1.76 µM. Compounds **3g** (3-OCH_3_-4-OH derivative) and **3e** (3,4-dihydroxy derivative) were the most potent pim-1 inhibitors in this study with IC_50_ of 0.08 and 0.06 µM, respectively. No significant difference was detected between the pim-1 inhibitory activity of the 4-pyrimidinone and the 4-imino (=NH) or the cyclised triazolopyrimidine derivatives. The most active compounds were tested for their cytotoxic activity on two cell lines [MCF7 and HCT116] using MTT method. All the compounds showed potent cytotoxic activity on both cell lines but compound **3e** [the 2–(3,4-dihydroxy) derivative] displayed the most potent cytotoxic effect on both cell lines with IC_50_ values of 0.22 and 0.37 µM and these results were consistent with its high kinase inhibitory activity (IC_50_ = 0.06 µM). A remarkable improvement in the cytotoxic activity was also noticed compared to the previously reported pyridothienopyrimidinones. Further work on pyridothienopyrimidine scaffold is still needed to obtain more potent pim-1 inhibitors and to improve the physicochemical properties of these derivatives.

## Supplementary Material

IENZ_1389921_Supplementary_Materials.pdf
