# Supplementary material for: Synthesis of new pyridothienopyrimidinone and pyridothienotriazolopyrimidine derivatives as pim-1 inhibitors
Source: J Enzyme Inhib Med Chem. 2017 Nov 21;33(1):58–66. doi: 10.1080/14756366.2017.1389921 (PMC6009955; doi:10.1080/14756366.2017.1389921)

Synthesis of New Pyridothienopyrimidinone and Pyridothienotriazolopyrimidine Derivatives as Pim-1 Inhibitors

Hala B. El-Nassan [1,*], Bassem H. Naguib [1,2], Engy A. Beshay [3]

Data File

Engy Alber_H_BO

Microanalytical Unit - FOPCU - NMR laboratory
www.pharma.cu.edu.eg dir-mau.fopcu@pharma.cu.edu.eg

BRUKER

Current Data Parameters
NAME Engy Alber_H_BO
EXPNO 10
PROCNO 1

F2 - Acquisition Parameters
Date_ 20170115
Time 22.23
INSTRUM spect
PROBHD 5 mm PABBO BB/
PULPROG zg30
TD 65536
SOLVENT DMSO
NS 32
DS 2
SWH 8012.820 Hz
FIDRES 0.122266 Hz
AQ 4.0894465 sec
RG 180.8
DW 62.400 usec
DE 6.50 usec
TE 298.1 K
D1 1.00000000 sec
TD0 1

======== CHANNEL f1 ========
SFO1 400.1924713 MHz
NUC1 1H
P1 15.00 usec
PLW1 10.39999962 W

F2 - Processing parameters
SI 65536
SF 400.1900000 MHz
WDW EM
SSB 0
LB 0.30 Hz
GB 0
PC 1.00

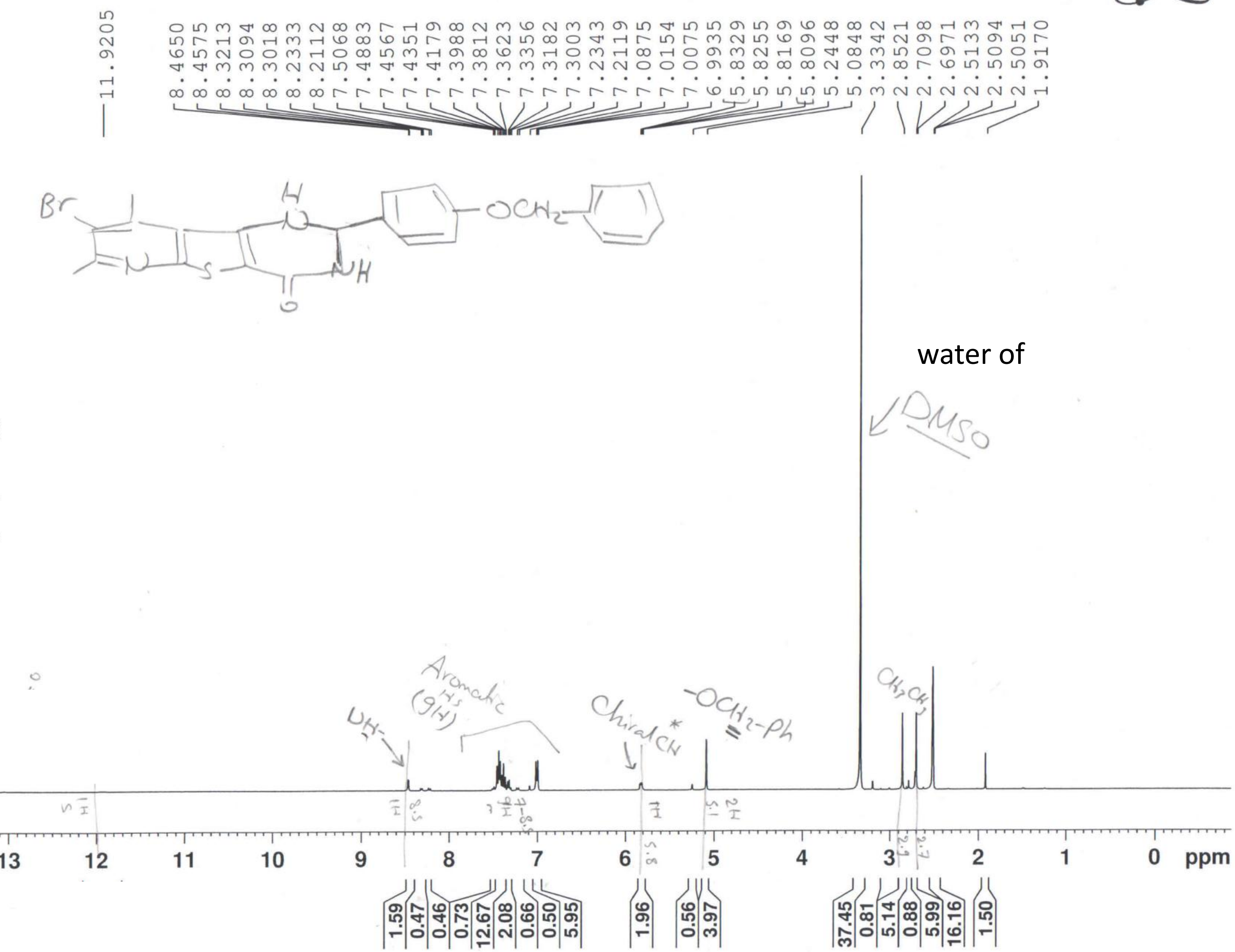

Engy Alber_H_4-Br

Microanalytical Unit - FOPCU - NMR laboratory
www.pharma.cu.edu.eg dir-mau.fopcu@pharma.cu.edu.eg

BRUKER

```
Current Data Parameters
NAME     Engy Alber_H_4-Br
EXPNO                   10
PROCNO                   1

F2 - Acquisition Parameters
Date_             20161104
Time                 13.23
INSTRUM              spect
PROBHD   5 mm PABBO BB/
PULPROG               zg30
TD                   65536
SOLVENT               DMSO
NS                      32
DS                       2
SWH            8012.820 Hz
FIDRES         0.122266 Hz
AQ            4.0894465 sec
RG                  169.46
DW              62.400 usec
DE                6.50 usec
TE                 298.1 K
D1          1.00000000 sec
TD0                      1

======== CHANNEL f1 ========
SFO1        400.1924713 MHz
NUC1                     1H
P1               15.00 usec
PLW1         10.39999962 W

F2 - Processing parameters
SI                   65536
SF          400.1900000 MHz
WDW                     EM
SSB      0
LB                 0.30 Hz
GB       0
PC                    1.00
```

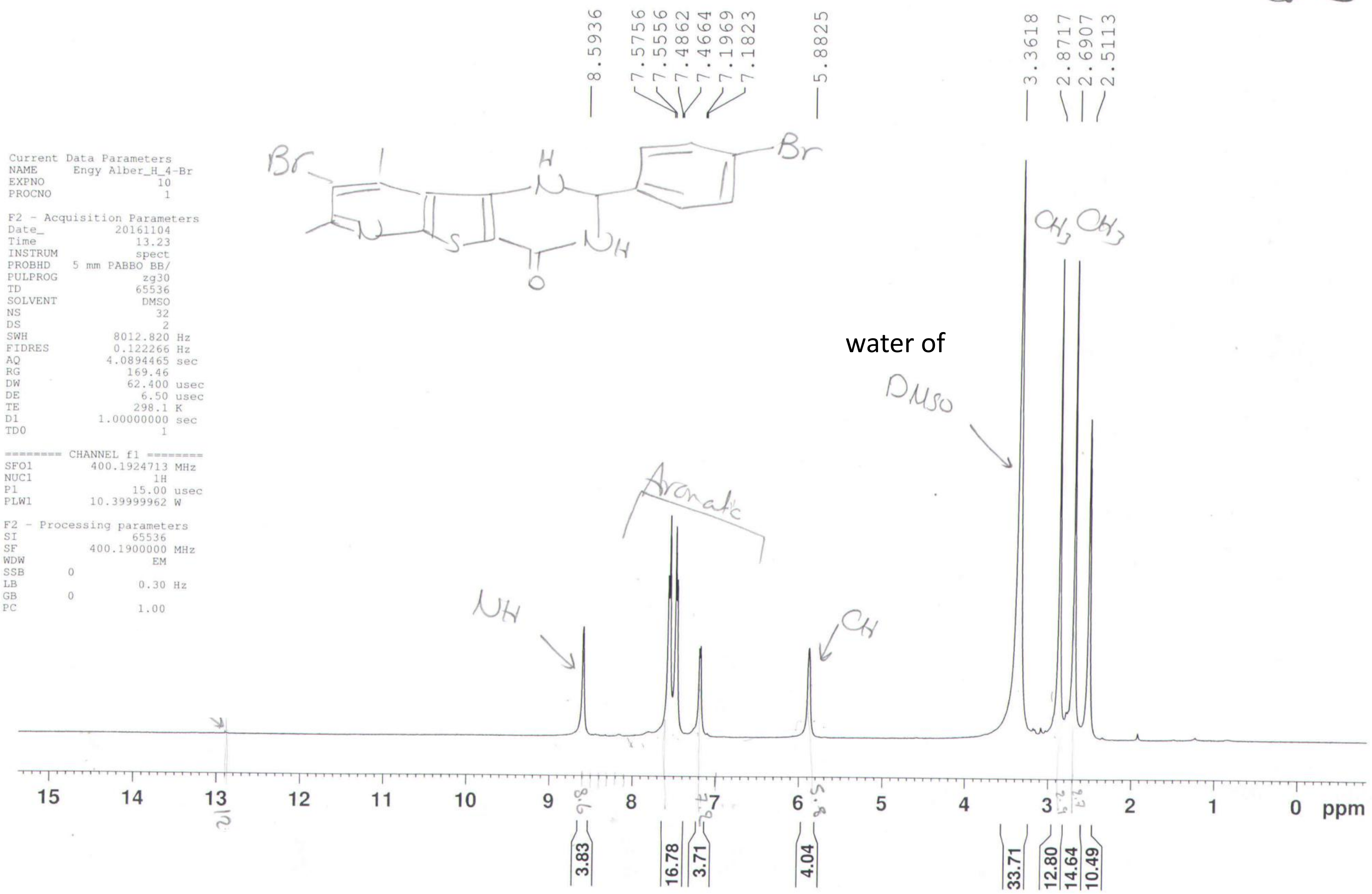

Engy Alber_C_4-Br

Microanalytical Unit - FOPCU - NMR laboratory
www.pharma.cu.edu.eg dir-mau.fopcu@pharma.cu.edu.eg

BRUKER

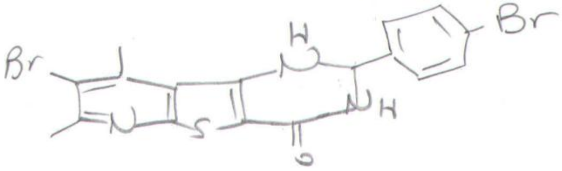

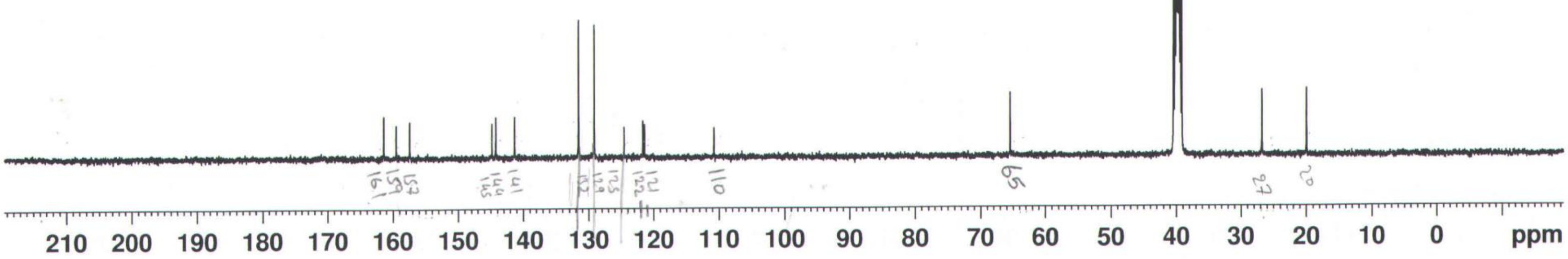


```
Current Data Parameters
NAME      Engy Alber_C_4-Br
EXPNO                    10
PROCNO                    1

F2 - Acquisition Parameters
Date_              20161212
Time                  10.43
INSTRUM               spect
PROBHD   5 mm PABBO BB/
PULPROG             zgpg30
TD                    65536
SOLVENT                DMSO
NS                     1200
DS                        4
SWH               24038.461 Hz
FIDRES             0.366798 Hz
AQ                1.3631488 sec
RG                   202.37
DW                   20.800 usec
DE                     6.50 usec
TE                    298.0 K
D1               2.00000000 sec
D11              0.03000000 sec
TD0                       1

======== CHANNEL f1 ========
SFO1            100.6379178 MHz
NUC1                    13C
P1                    10.00 usec
PLW1            45.00000000 W

======== CHANNEL f2 ========
SFO2            400.1916008 MHz
NUC2                     1H
CPDPRG[2            waltz16
```

Ingi Alber_H_P-Cl

Microanalytical Unit - FOPCU - NMR laboratory
www.pharma.cu.edu.eg dir-mau.fopcu@pharma.cu.edu.eg

BRUKER

Current Data Parameters
NAME Ingi Alber_H_P-Cl
EXPNO 40
PROCNO 1

F2 - Acquisition Parameters
Date_ 20160815
Time 12.25
INSTRUM spect
PROBHD 5 mm PABBO BB/
PULPROG zg30
TD 65536
SOLVENT DMSO
NS 32
DS 2
SWH 8012.820 Hz
FIDRES 0.122266 Hz
AQ 4.0894465 sec
RG 180.8
DW 62.400 usec
DE 6.50 usec
TE 298.0 K
D1 1.00000000 sec
TD0 1

======== CHANNEL f1 ========
SFO1 400.1924713 MHz
NUC1 1H
P1 15.00 usec
PLW1 10.39999962 W

F2 - Processing parameters
SI 65536
SF 400.1900000 MHz
WDW EM
SSB 0
LB 0.30 Hz
GB 0
PC 1.00

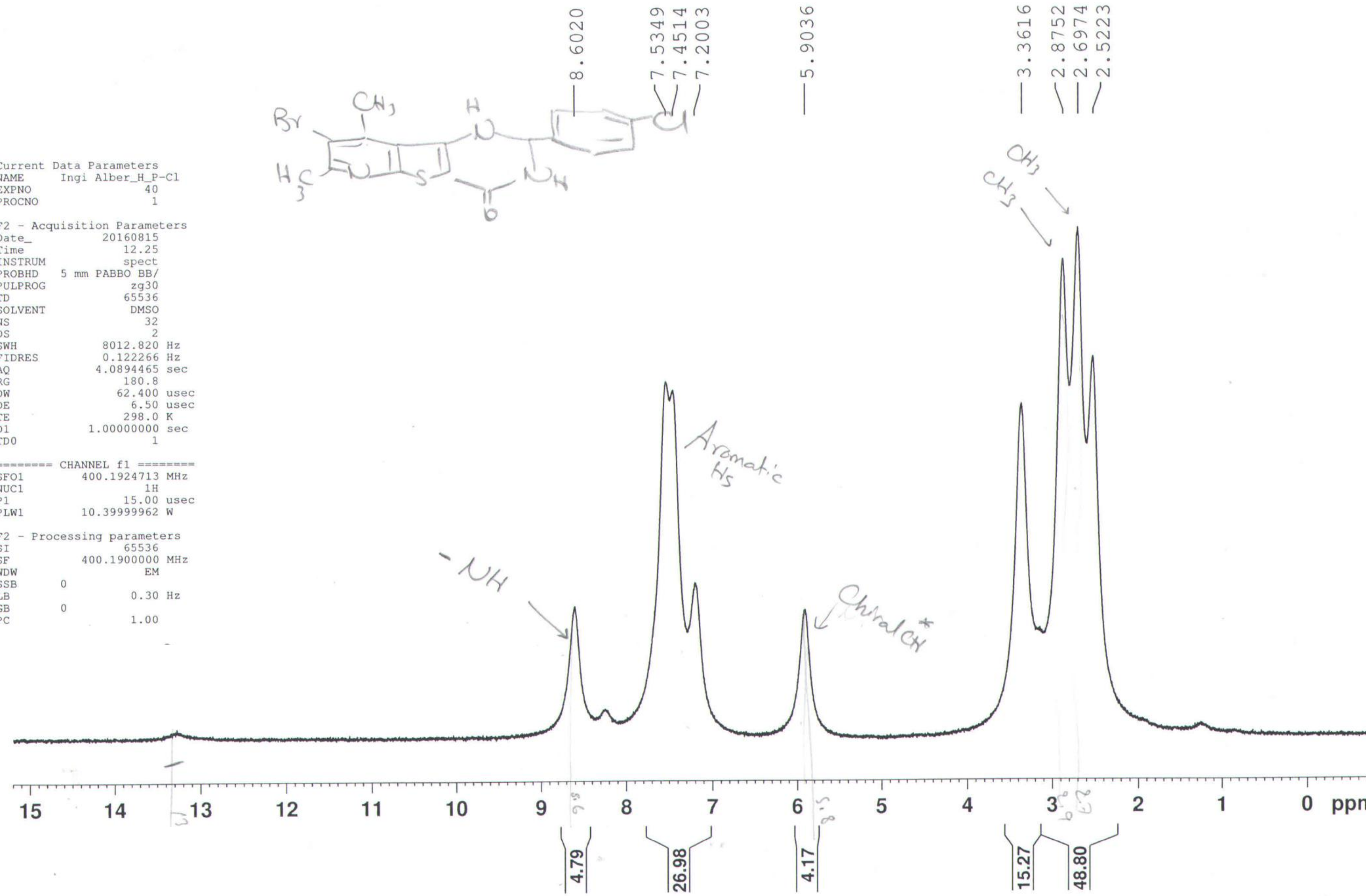

Engy Alber_H_2,4 diOH

Microanalytical Unit - FOPCU - NMR laboratory
www.pharma.cu.edu.eg dir-mau.fopcu@pharma.cu.edu.eg

BRUKER

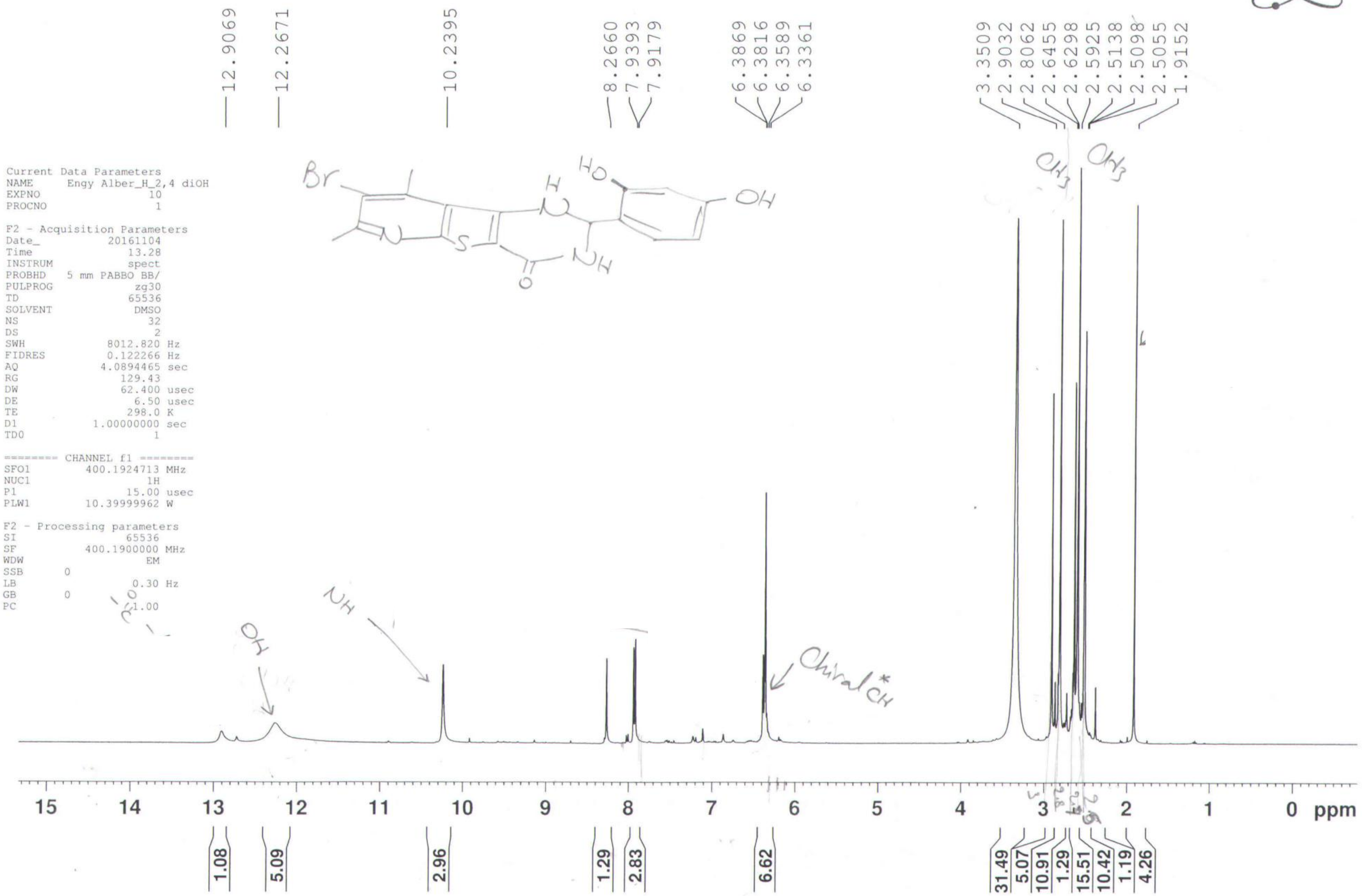

Current Data Parameters
NAME Engy Alber_H_2,4 diOH
EXPNO 10
PROCNO 1

F2 - Acquisition Parameters
Date_ 20161104
Time 13.28
INSTRUM spect
PROBHD 5 mm PABBO BB/
PULPROG zg30
TD 65536
SOLVENT DMSO
NS 32
DS 2
SWH 8012.820 Hz
FIDRES 0.122266 Hz
AQ 4.0894465 sec
RG 129.43
DW 62.400 usec
DE 6.50 usec
TE 298.0 K
D1 1.00000000 sec
TD0 1

======== CHANNEL f1 ========
SFO1 400.1924713 MHz
NUC1 1H
P1 15.00 usec
PLW1 10.39999962 W

F2 - Processing parameters
SI 65536
SF 400.1900000 MHz
WDW EM
SSB 0
LB 0.30 Hz
GB 0
PC 1.00

Engy Alber_C_3,4 diOH

Microanalytical Unit - FOPCU - NMR laboratory
www.pharma.cu.edu.eg dir-mau.fopcu@pharma.cu.edu.eg

BRUKER

```
Current Data Parameters
NAME        Engy Alber_C_3,4 diOH
EXPNO                          10
PROCNO                          1

F2 - Acquisition Parameters
Date_                    20161205
Time                        15.52
INSTRUM                      spect
PROBHD      5 mm PABBO BB/
PULPROG                    zgpg30
TD                          65536
SOLVENT                      DMSO
NS                           1200
DS                              4
SWH                 24038.461 Hz
FIDRES               0.366798 Hz
AQ                  1.3631488 sec
RG                         202.37
DW                     20.800 usec
DE                       6.50 usec
TE                        298.0 K
D1                 2.00000000 sec
D11                0.03000000 sec
TD0                             1

======== CHANNEL f1 ========
SFO1              100.6379178 MHz
NUC1                          13C
P1                      10.00 usec
PLW1              45.00000000 W

======== CHANNEL f2 ========
SFO2              400.1916008 MHz
NUC2                           1H
CPDPRG[2                  waltz16
```

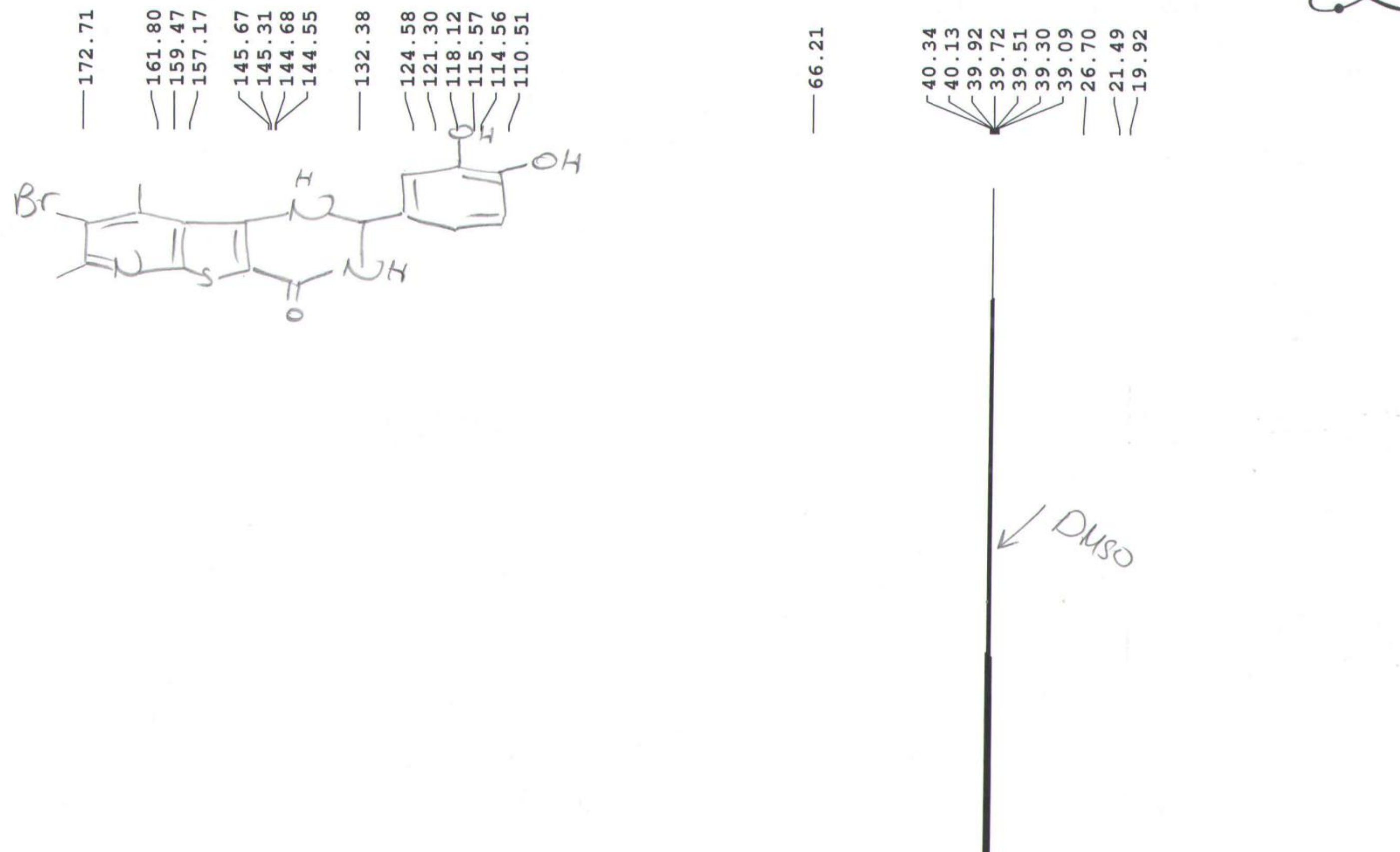

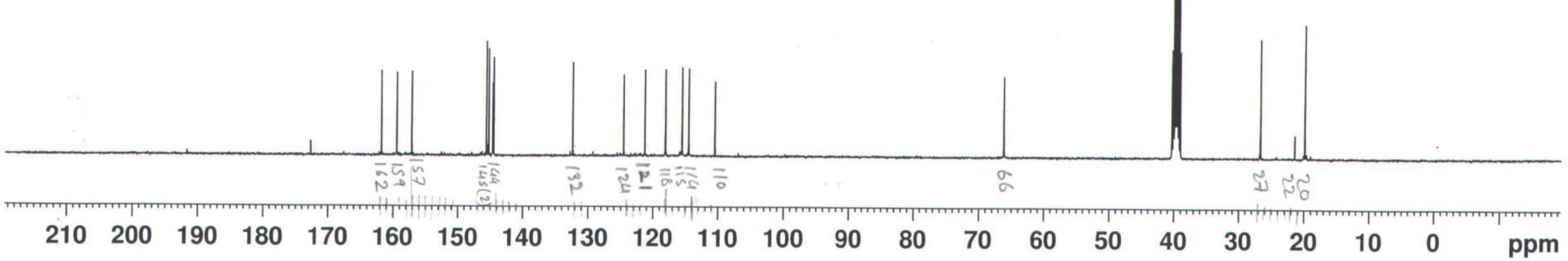

Engy Alber_H_3,4 diOH

Microanalytical Unit - FOPCU - NMR laboratory
www.pharma.cu.edu.eg dir-mau.fopcu@pharma.cu.edu.eg

BRUKER

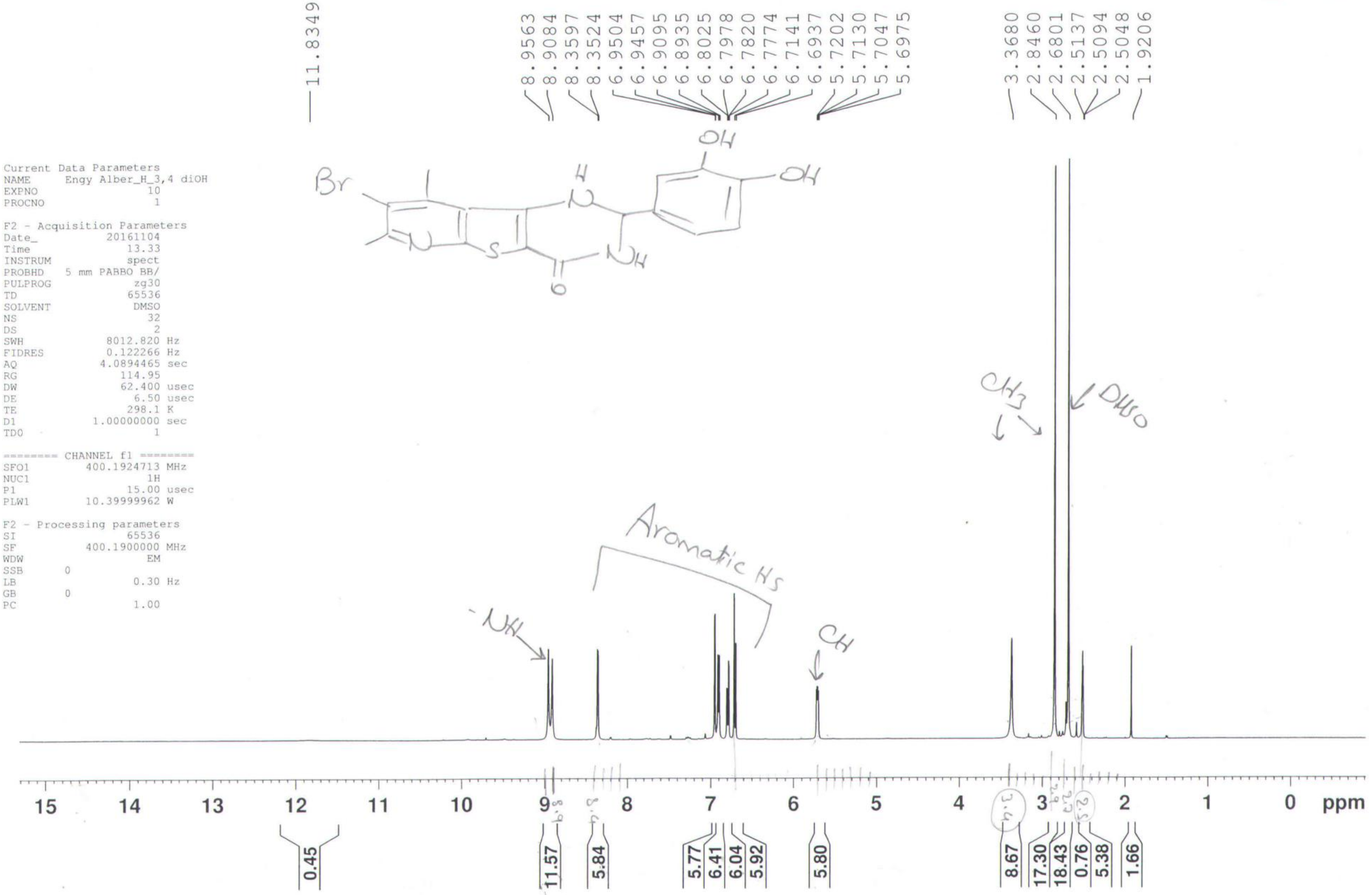


```
Current Data Parameters
NAME       Engy Alber_H_3,4 diOH
EXPNO                 10
PROCNO                 1

F2 - Acquisition Parameters
Date_           20161104
Time               13.33
INSTRUM            spect
PROBHD     5 mm PABBO BB/
PULPROG             zg30
TD                 65536
SOLVENT             DMSO
NS                    32
DS                     2
SWH            8012.820 Hz
FIDRES         0.122266 Hz
AQ            4.0894465 sec
RG                114.95
DW                62.400 usec
DE                  6.50 usec
TE                 298.1 K
D1            1.00000000 sec
TD0                    1

======== CHANNEL f1 ========
SFO1         400.1924713 MHz
NUC1                  1H
P1                 15.00 usec
PLW1         10.39999962 W

F2 - Processing parameters
SI                 65536
SF           400.1900000 MHz
WDW                   EM
SSB        0
LB                  0.30 Hz
GB         0
PC                  1.00
```

Engy Alber_H_4-F

Microanalytical Unit - FOPCU - NMR laboratory
www.pharma.cu.edu.eg dir-mau.fopcu@pharma.cu.edu.eg

BRUKER

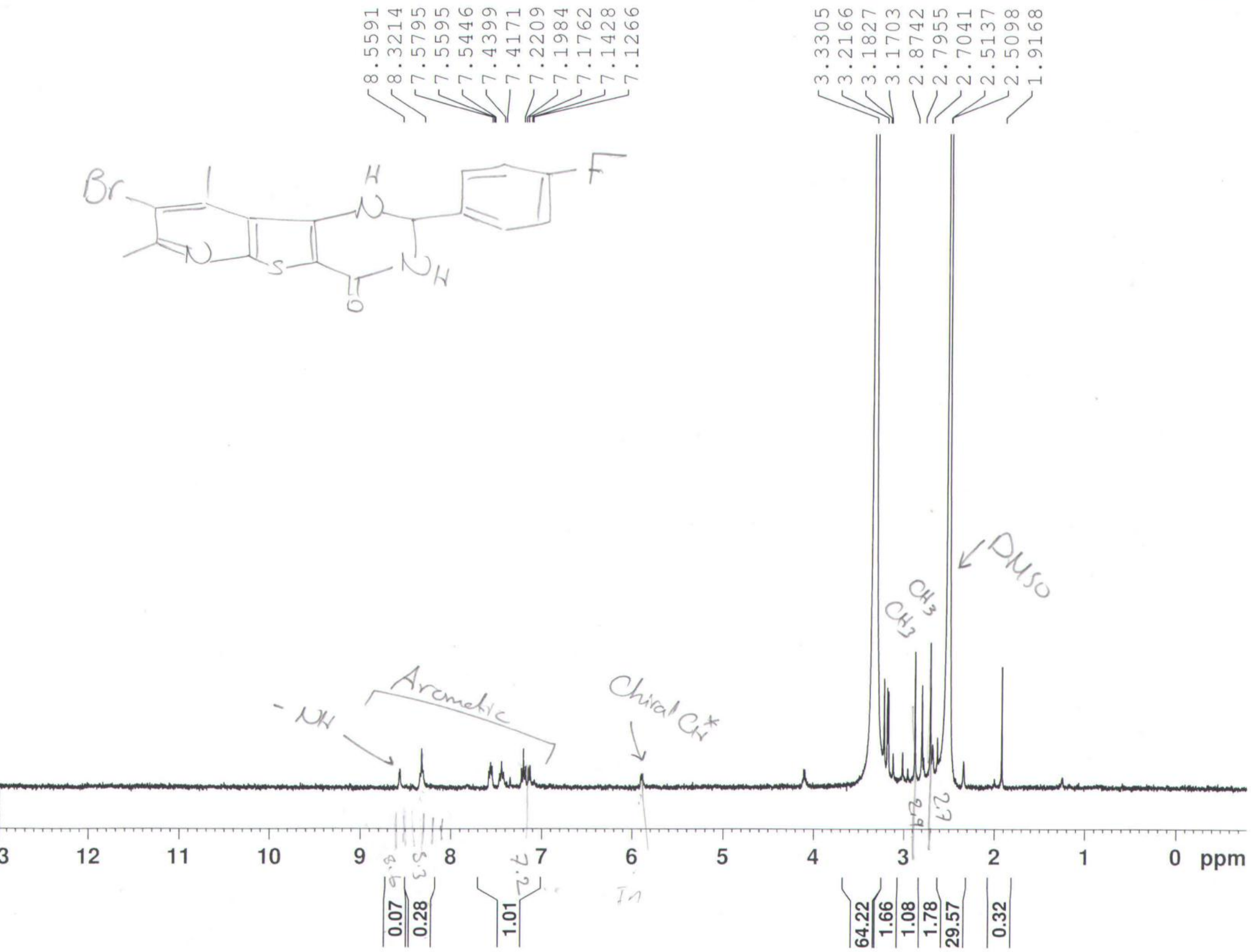


```
Current Data Parameters
NAME      Engy Alber_H_4-F
EXPNO                   10
PROCNO                   1

F2 - Acquisition Parameters
Date_            20161104
Time                13.18
INSTRUM             spect
PROBHD   5 mm PABBO BB/
PULPROG              zg30
TD                  65536
SOLVENT              DMSO
NS                     32
DS                      2
SWH              8012.820 Hz
FIDRES           0.122266 Hz
AQ              4.0894465 sec
RG                 202.37
DW                 62.400 usec
DE                   6.50 usec
TE                  298.1 K
D1             1.00000000 sec
TD0                     1

======== CHANNEL f1 ========
SFO1          400.1924713 MHz
NUC1                   1H
P1                  15.00 usec
PLW1          10.39999962 W

F2 - Processing parameters
SI                  65536
SF            400.1900000 MHz
WDW                    EM
SSB      0
LB                   0.30 Hz
GB       0
PC                   1.00
```

Ingi Alber_H_Va

Microanalytical Unit - FOPCU - NMR laboratory
www.pharma.cu.edu.eg dir-mau.fopcu@pharma.cu.edu.eg

BRUKER

```
Current Data Parameters
NAME        Ingi Alber_H_Va
EXPNO                    20
PROCNO                    1

F2 - Acquisition Parameters
Date_              20160815
Time                  12.36
INSTRUM               spect
PROBHD    5 mm PABBO BB/
PULPROG                zg30
TD                    65536
SOLVENT                DMSO
NS                       32
DS                        2
SWH             8012.820 Hz
FIDRES          0.122266 Hz
AQ             4.0894465 sec
RG                   202.37
DW               62.400 usec
DE                 6.50 usec
TE                298.0 K
D1           1.00000000 sec
TD0                       1

======== CHANNEL f1 ========
SFO1       400.1924713 MHz
NUC1                    1H
P1                15.00 usec
PLW1        10.39999962 W

F2 - Processing parameters
SI                    65536
SF         400.1900000 MHz
WDW                      EM
SSB       0
LB                  0.30 Hz
GB        0
PC                     1.00
```

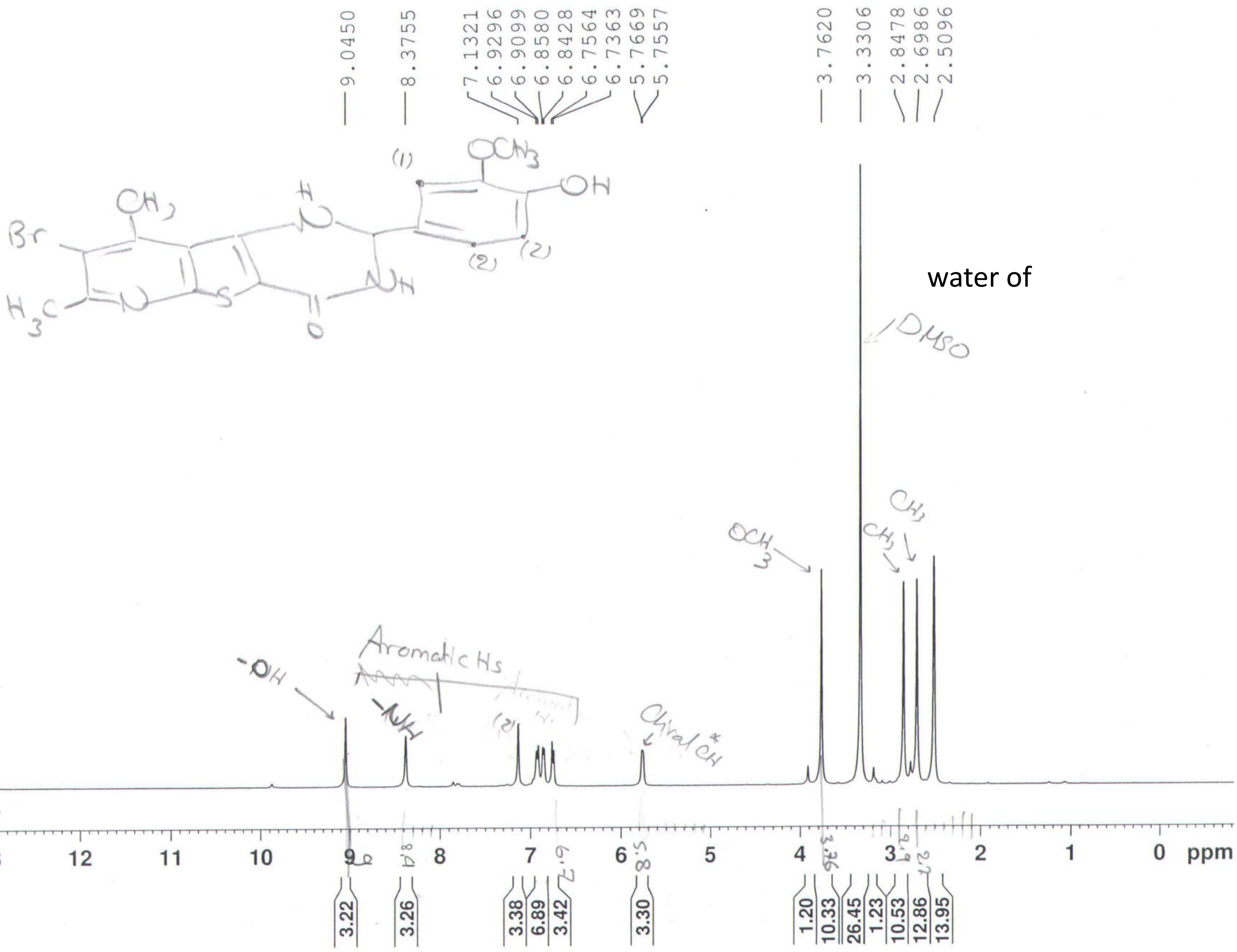

Engy Alber_C_Va

Microanalytical Unit - FOPCU - NMR laboratory
www.pharma.cu.edu.eg dir-mau.fopcu@pharma.cu.edu.eg

BRUKER

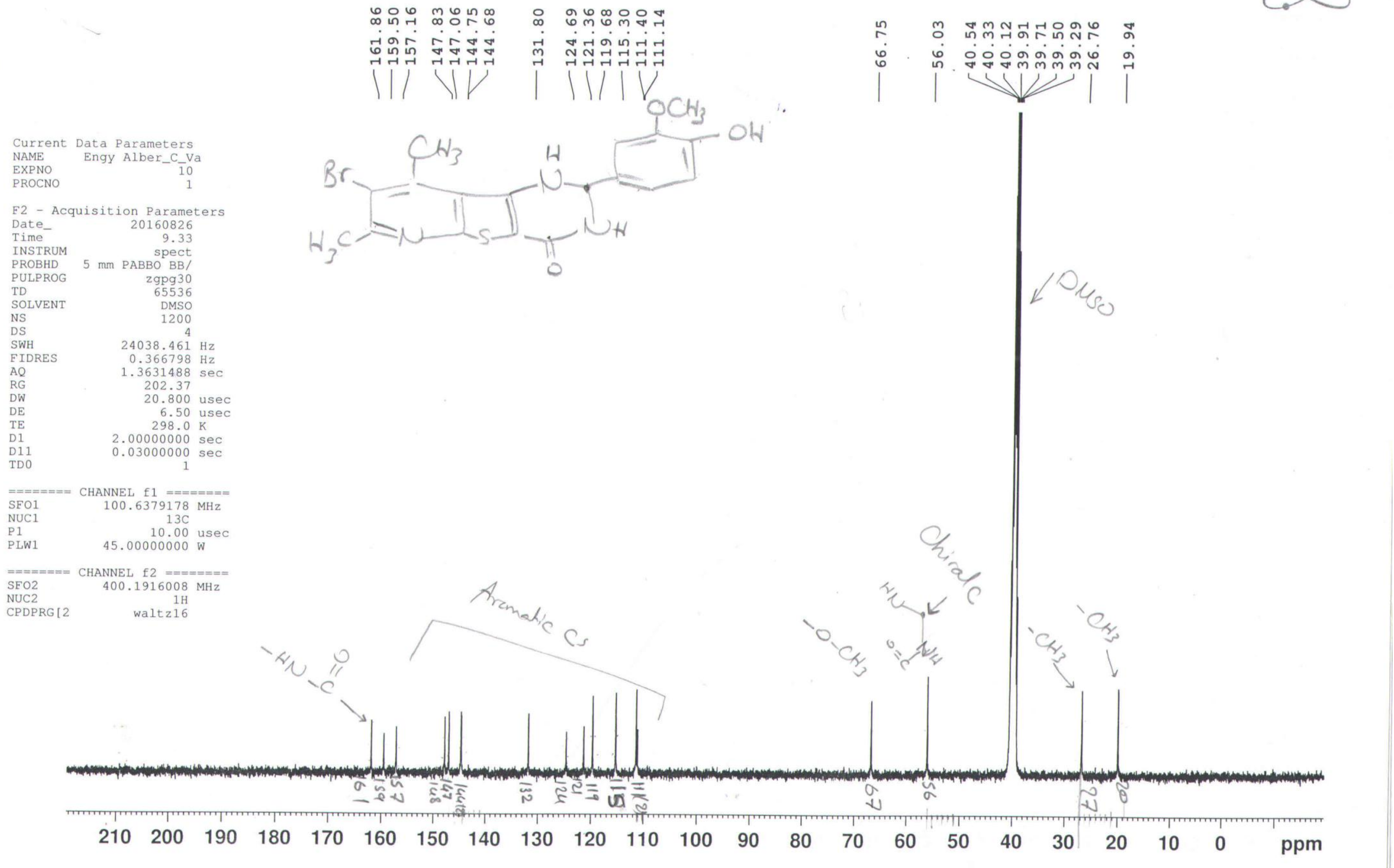

Ingi Alber_H_4-MeO

Microanalytical Unit - FOPCU - NMR laboratory
www.pharma.cu.edu.eg dir-mau.fopcu@pharma.cu.edu.eg

BRUKER

```
Current Data Parameters
NAME      Ingi Alber_H_4-MeO
EXPNO                     20
PROCNO                     1

F2 - Acquisition Parameters
Date_             20160815
Time                 12.41
INSTRUM              spect
PROBHD   5 mm PABBO BB/
PULPROG               zg30
TD                   65536
SOLVENT               DMSO
NS                      32
DS                       2
SWH               8012.820 Hz
FIDRES            0.122266 Hz
AQ               4.0894465 sec
RG                  202.37
DW                  62.400 usec
DE                    6.50 usec
TE                   298.0 K
D1              1.00000000 sec
TD0                      1

======== CHANNEL f1 ========
SFO1         400.1924713 MHz
NUC1                    1H
P1                   15.00 usec
PLW1           10.39999962 W

F2 - Processing parameters
SI                   65536
SF             400.1900000 MHz
WDW                     EM
SSB      0
LB                    0.30 Hz
GB       0
PC                    1.00
```

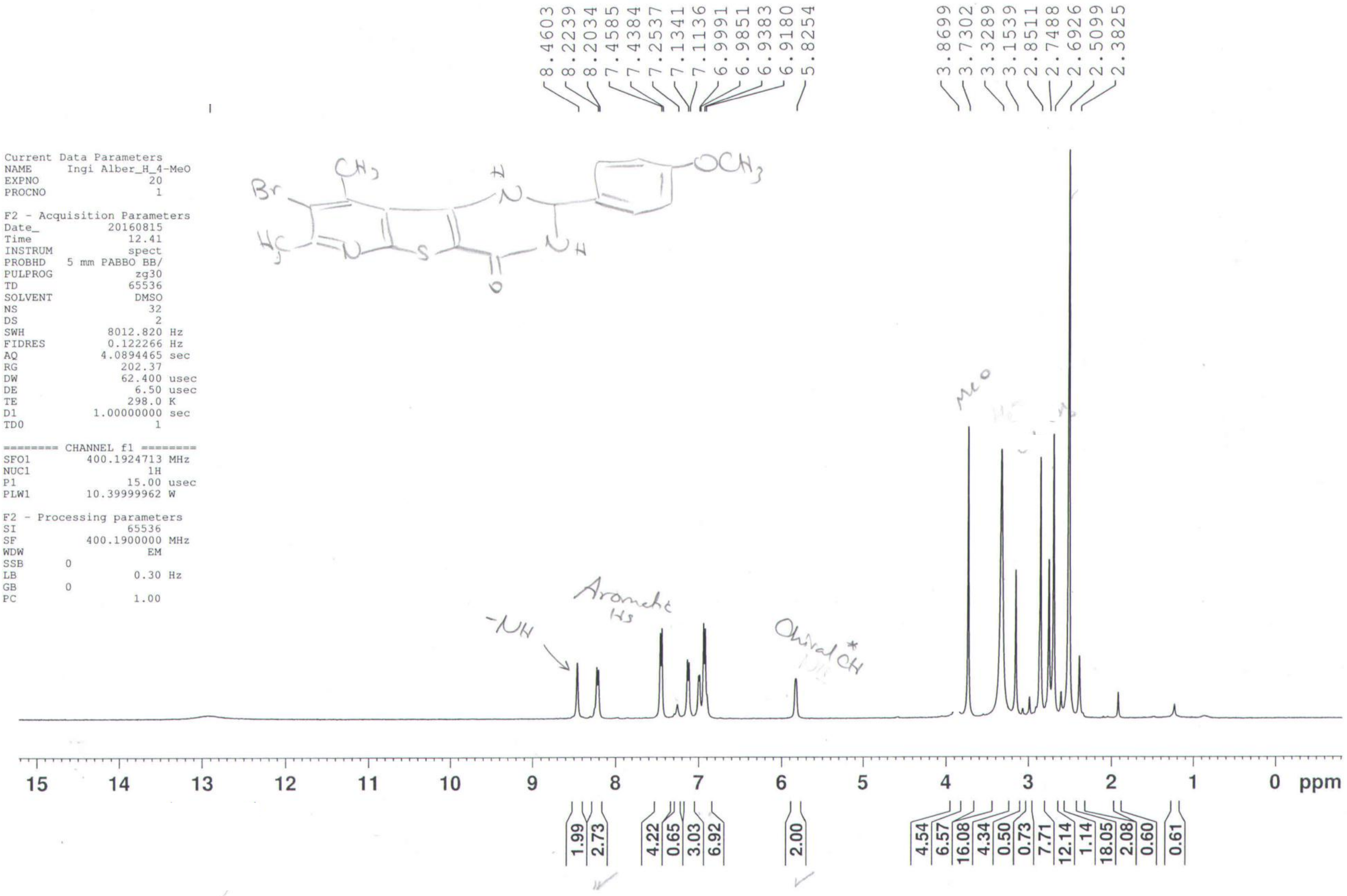

Tri MeO

Ingi Alber_H_3MeO

Microanalytical Unit - FOPCU - NMR laboratory
www.pharma.cu.edu.eg dir-mau.fopcu@pharma.cu.edu.eg

BRUKER

Current Data Parameters
NAME Ingi Alber_H_3MeO
EXPNO 20
PROCNO 1

F2 - Acquisition Parameters
Date_ 20160815
Time 12.31
INSTRUM spect
PROBHD 5 mm PABBO BB/
PULPROG zg30
TD 65536
SOLVENT DMSO
NS 32
DS 2
SWH 8012.820 Hz
FIDRES 0.122266 Hz
AQ 4.0894465 sec
RG 169.46
DW 62.400 usec
DE 6.50 usec
TE 298.0 K
D1 1.00000000 sec
TD0 1

======== CHANNEL f1 ========
SFO1 400.1924713 MHz
NUC1 1H
P1 15.00 usec
PLW1 10.39999962 W

F2 - Processing parameters
SI 65536
SF 400.1900000 MHz
WDW EM
SSB 0
LB 0.30 Hz
GB 0
PC 1.00

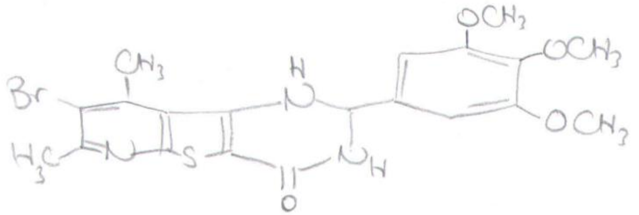

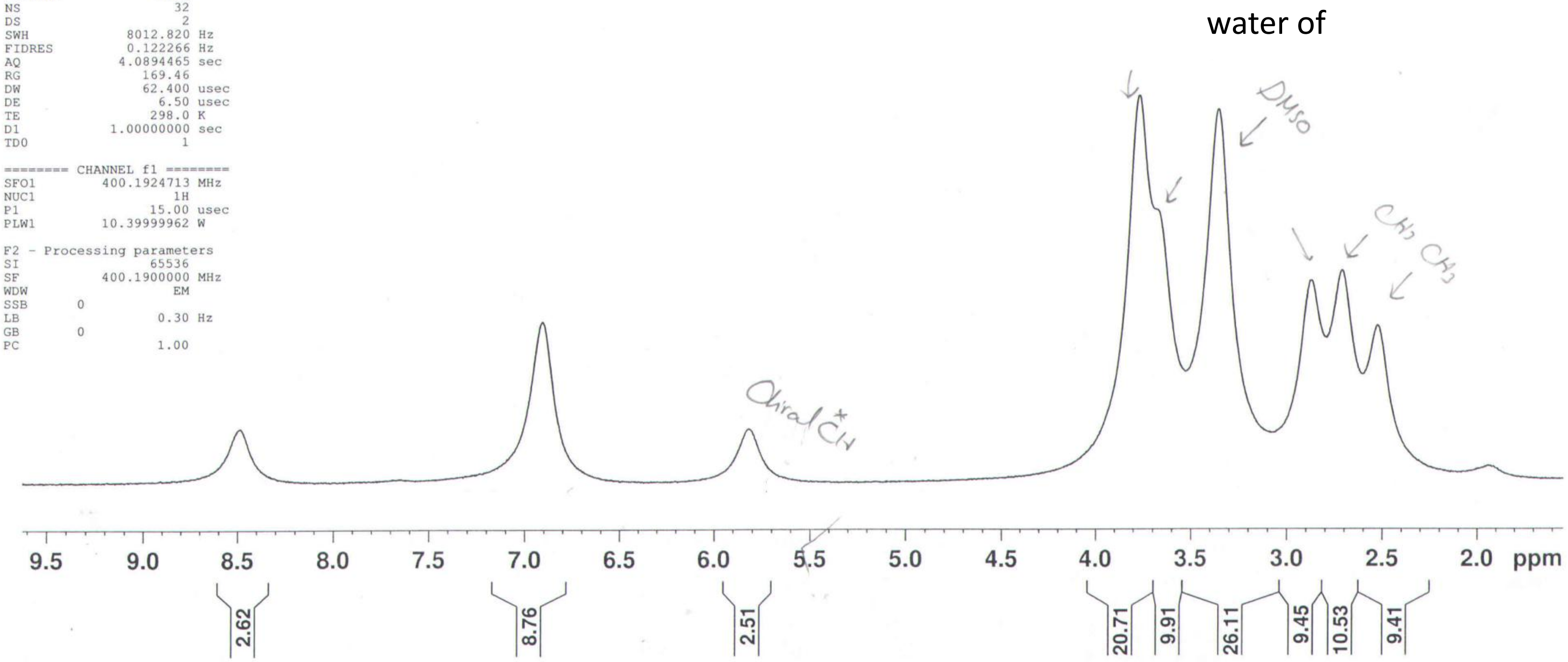

Engy Alber_H_T3A

Microanalytical Unit - FOPCU - NMR laboratory
www.pharma.cu.edu.eg dir-mau.fopcu@pharma.cu.edu.eg

BRUKER

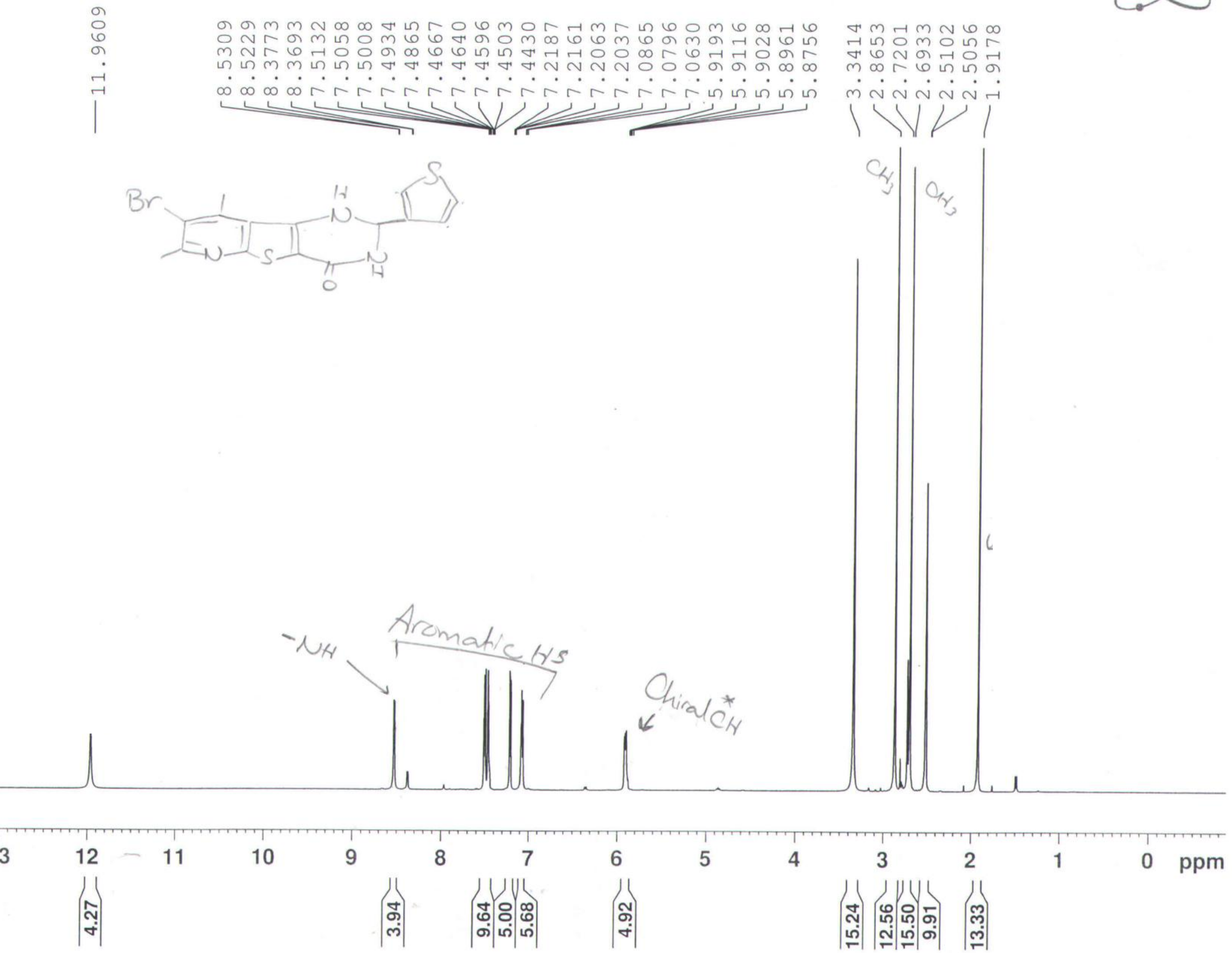

Current Data Parameters
NAME Engy Alber_H_T3A
EXPNO 10
PROCNO 1

F2 - Acquisition Parameters
Date_ 20161019
Time 15.39
INSTRUM spect
PROBHD 5 mm PABBO BB/
PULPROG zg30
TD 65536
SOLVENT DMSO
NS 32
DS 2
SWH 8012.820 Hz
FIDRES 0.122266 Hz
AQ 4.0894465 sec
RG 146.06
DW 62.400 usec
DE 6.50 usec
TE 298.1 K
D1 1.00000000 sec
TD0 1

======== CHANNEL f1 ========
SFO1 400.1924713 MHz
NUC1 1H
P1 15.00 usec
PLW1 10.39999962 W

F2 - Processing parameters
SI 65536
SF 400.1900000 MHz
WDW EM
SSB 0
LB 0.30 Hz
GB 0
PC 1.00

Engy Alber_C_T3A

Microanalytical Unit - FOPCU - NMR laboratory
www.pharma.cu.edu.eg dir-mau.fopcu@pharma.cu.edu.eg

BRUKER

```
Current Data Parameters
NAME        Engy Alber_C_T3A
EXPNO                     10
PROCNO                     1

F2 - Acquisition Parameters
Date_               20161104
Time                   15.01
INSTRUM                spect
PROBHD     5 mm PABBO BB/
PULPROG              zgpg30
TD                     65536
SOLVENT                 DMSO
NS                      1500
DS                         4
SWH            24038.461 Hz
FIDRES          0.366798 Hz
AQ             1.3631488 sec
RG                    202.37
DW                20.800 usec
DE                  6.50 usec
TE                   298.1 K
D1            2.00000000 sec
D11           0.03000000 sec
TD0                        1

======== CHANNEL f1 ========
SFO1        100.6379178 MHz
NUC1                     13C
P1                 10.00 usec
PLW1         45.00000000 W

======== CHANNEL f2 ========
SFO2        400.1916008 MHz
NUC2                      1H
CPDPRG[2             waltz16
```

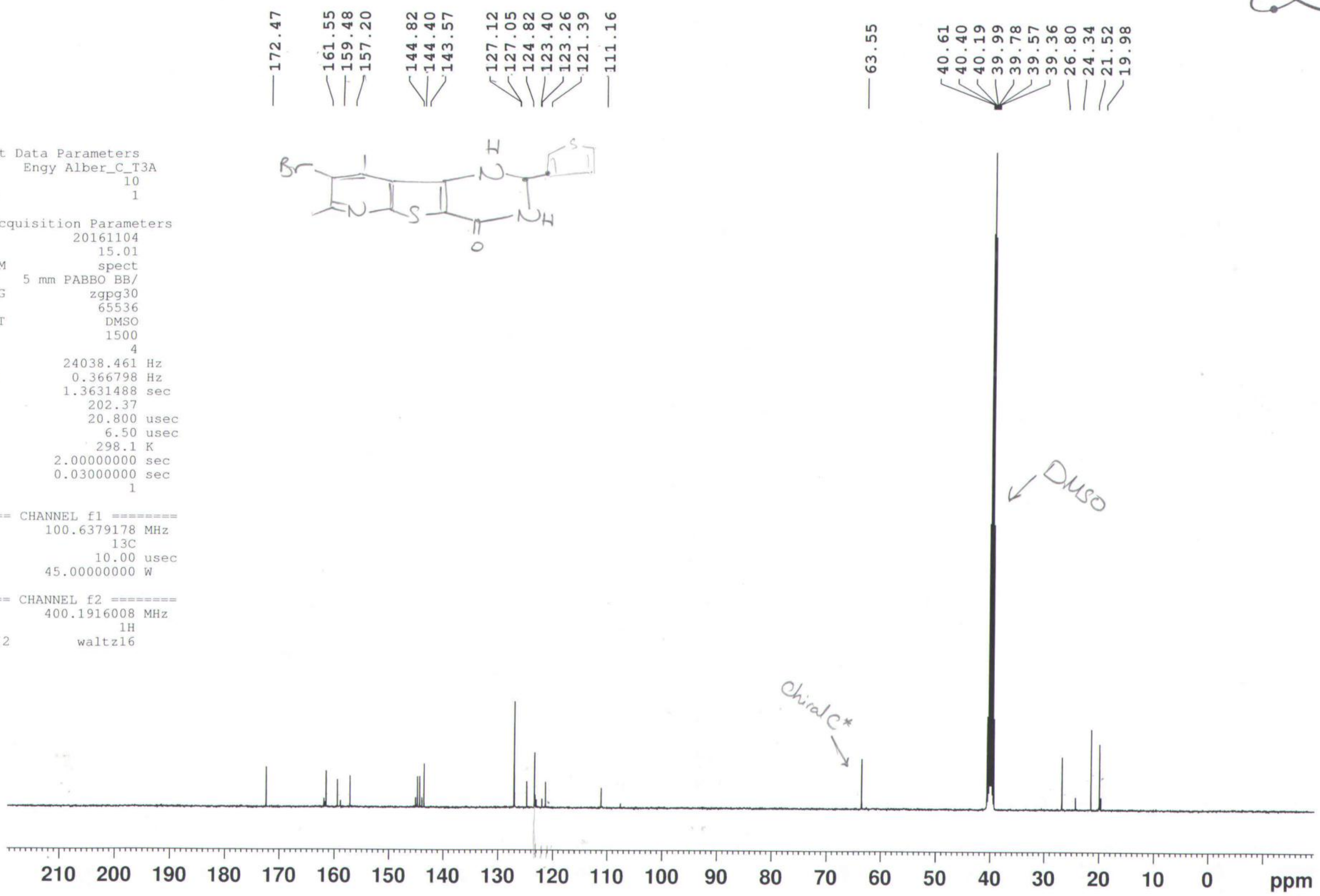

Engy Alber_H_2a

Microanalytical Unit - FOPCU - NMR laboratory
www.pharma.cu.edu.eg dir-mau.fopcu@pharma.cu.edu.eg

```
Current Data Parameters
NAME     Engy Alber_H_2a
EXPNO                 10
PROCNO                 1

F2 - Acquisition Parameters
Date_           20170228
Time               16.31
INSTRUM            spect
PROBHD   5 mm PABBO BB/
PULPROG             zg30
TD                 65536
SOLVENT             DMSO
NS                    32
DS                     2
SWH          8012.820 Hz
FIDRES       0.122266 Hz
AQ          4.0894465 sec
RG                146.06
DW            62.400 usec
DE              6.50 usec
TE               298.1 K
D1        1.00000000 sec
TD0                    1

======== CHANNEL f1 ========
SFO1      400.1924713 MHz
NUC1                  1H
P1             15.00 usec
PLW1      10.39999962 W

F2 - Processing parameters
SI                 65536
SF        400.1900000 MHz
WDW                   EM
SSB      0
LB               0.30 Hz
GB       0
PC                  1.00
```

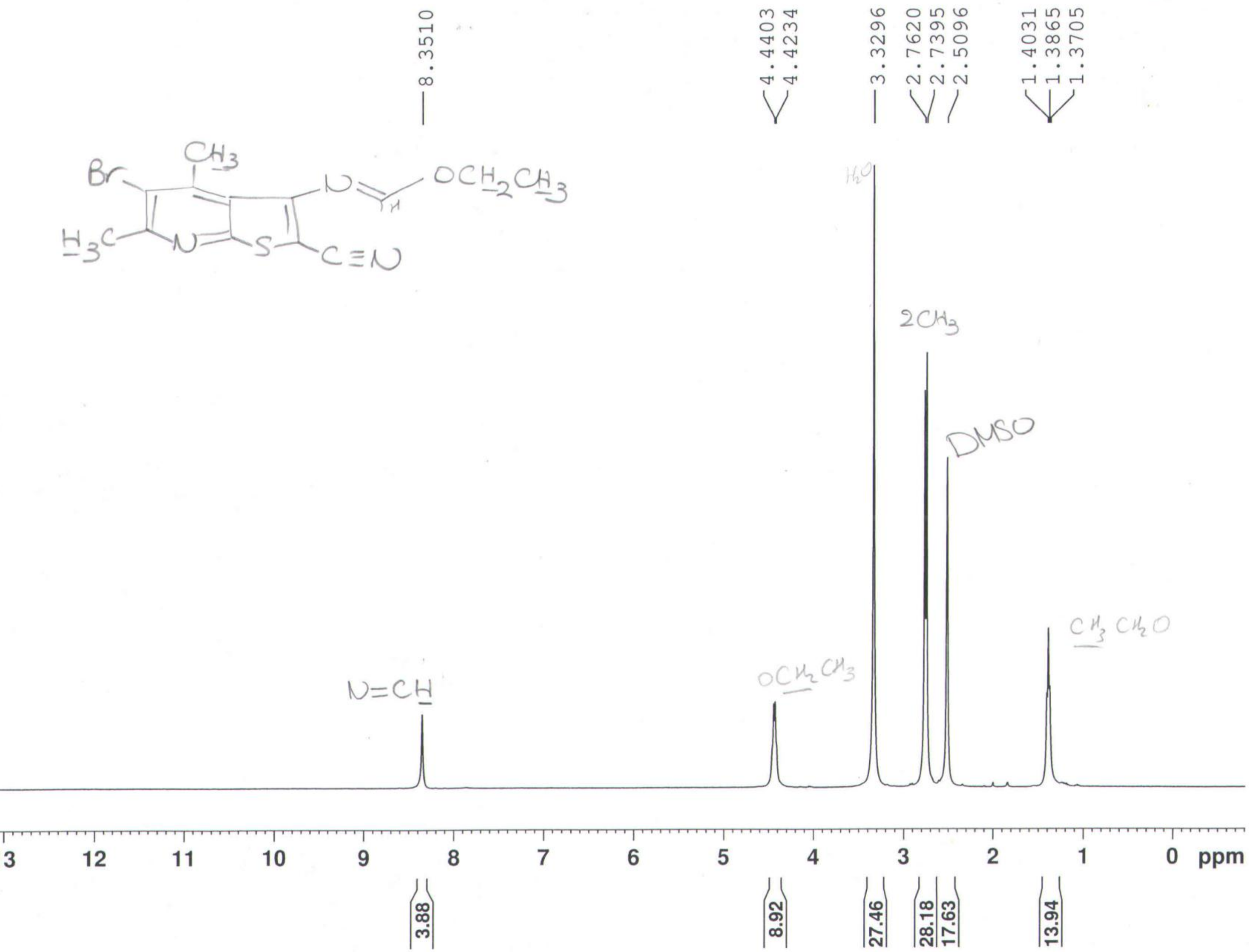

Engy Alber_H_22

Microanalytical Unit - FOPCU - NMR laboratory
www.pharma.cu.edu.eg dir-mau.fopcu@pharma.cu.edu.eg

BRUKER

Start of Scheme (2)

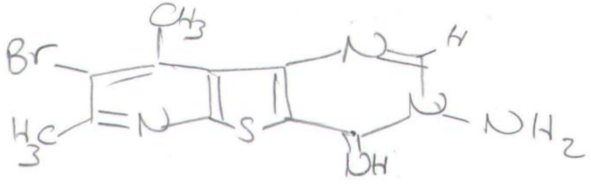

Current Data Parameters
NAME Engy Alber_H_22
EXPNO 10
PROCNO 1

F2 - Acquisition Parameters
Date_ 20170214
Time 16.34
INSTRUM spect
PROBHD 5 mm PABBO BB/
PULPROG zg30
TD 65536
SOLVENT DMSO
NS 32
DS 2
SWH 8012.820 Hz
FIDRES 0.122266 Hz
AQ 4.0894465 sec
RG 202.37
DW 62.400 usec
DE 6.50 usec
TE 298.1 K
D1 1.00000000 sec
TD0 1

======== CHANNEL f1 ========
SFO1 400.1924713 MHz
NUC1 1H
P1 15.00 usec
PLW1 10.39999962 W

F2 - Processing parameters
SI 65536
SF 400.1900000 MHz
WDW EM
SSB 0
LB 0.30 Hz
GB 0
PC 1.00

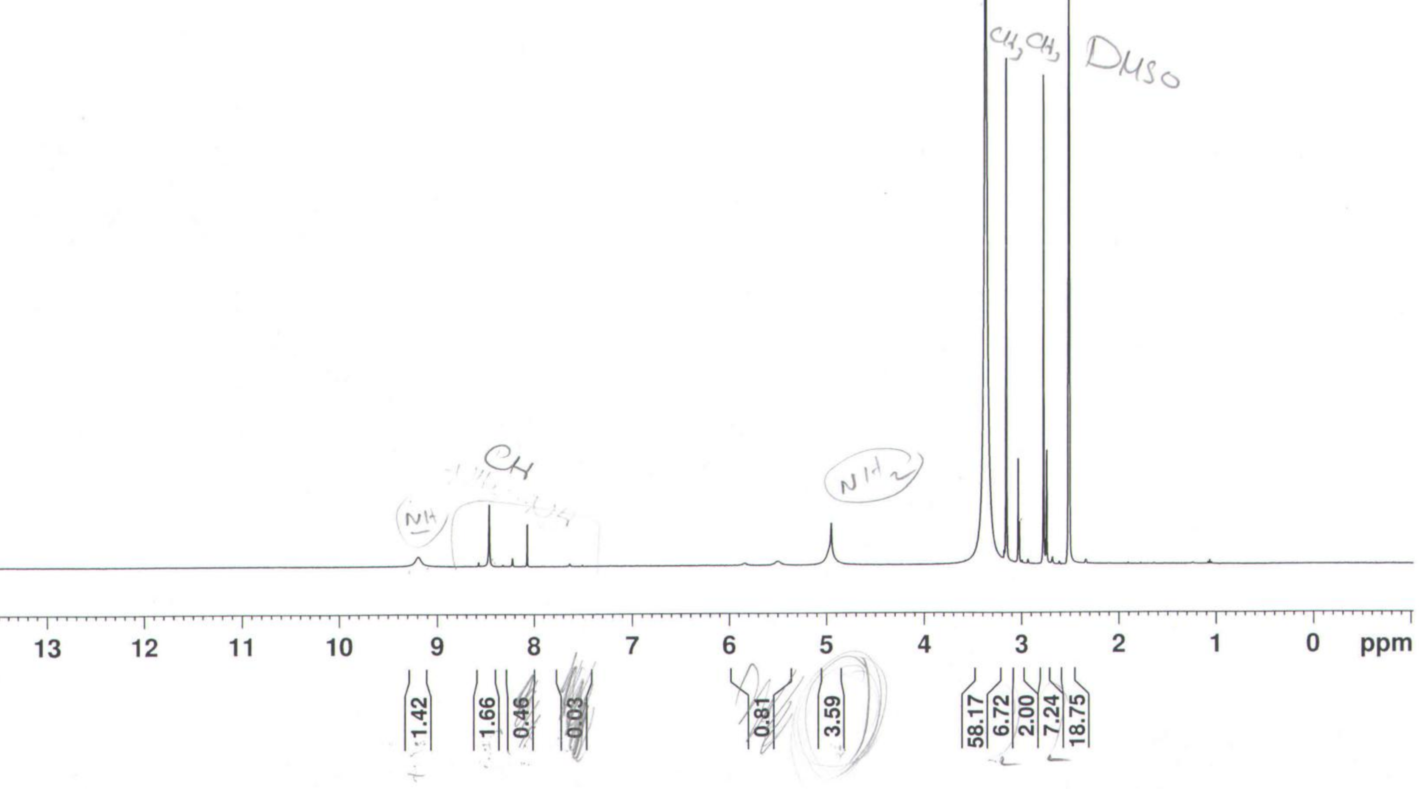

Engy Alber_H_2-5

Microanalytical Unit - FOPCU - NMR laboratory
www.pharma.cu.edu.eg dir-mau.fopcu@pharma.cu.edu.eg

BRUKER

Formicacid

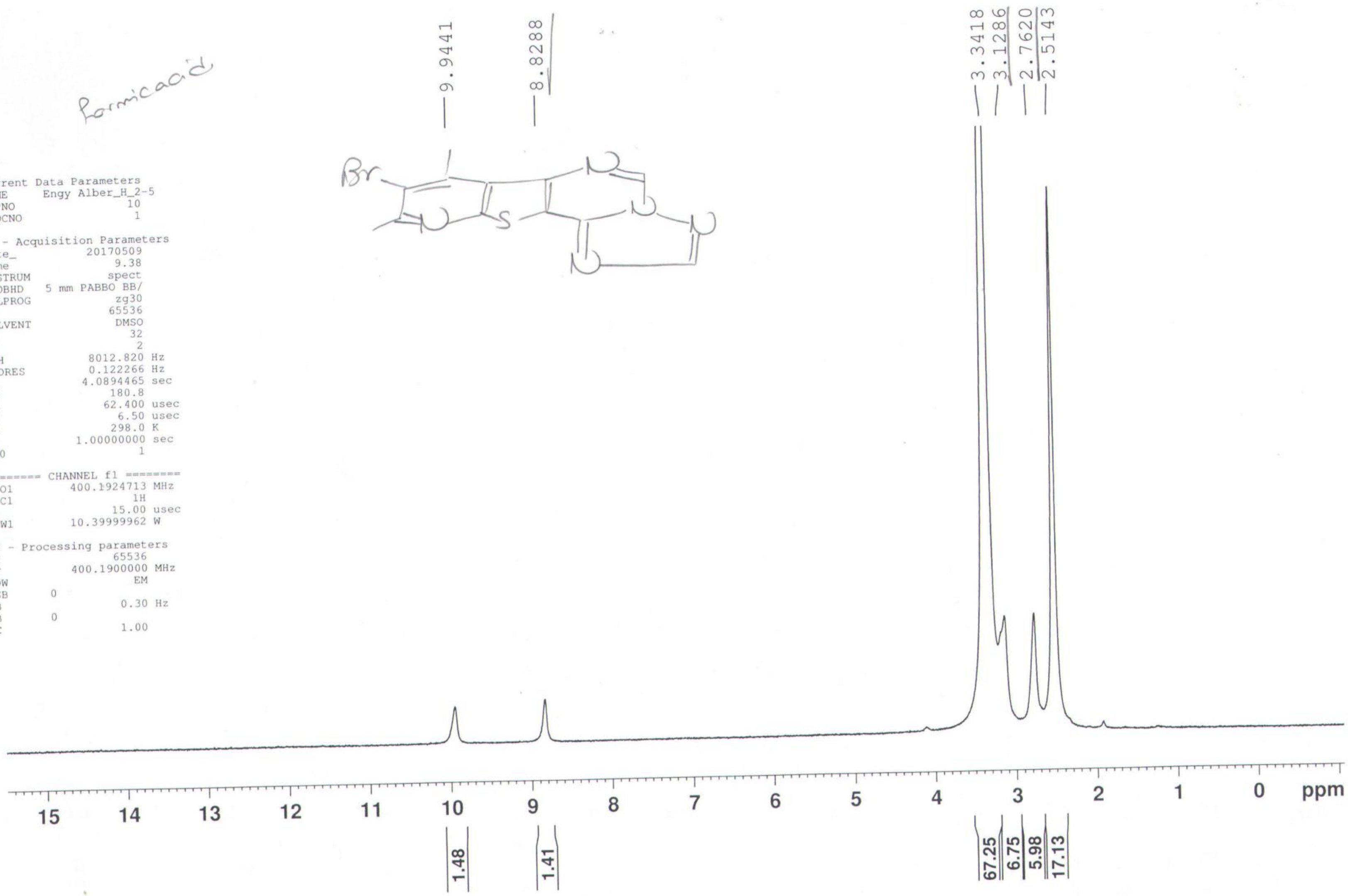

Current Data Parameters
NAME Engy Alber_H_2-5
EXPNO 10
PROCNO 1

F2 - Acquisition Parameters
Date_ 20170509
Time 9.38
INSTRUM spect
PROBHD 5 mm PABBO BB/
PULPROG zg30
TD 65536
SOLVENT DMSO
NS 32
DS 2
SWH 8012.820 Hz
FIDRES 0.122266 Hz
AQ 4.0894465 sec
RG 180.8
DW 62.400 usec
DE 6.50 usec
TE 298.0 K
D1 1.00000000 sec
TD0 1

======== CHANNEL f1 ========
SFO1 400.1924713 MHz
NUC1 1H
P1 15.00 usec
PLW1 10.39999962 W

F2 - Processing parameters
SI 65536
SF 400.1900000 MHz
WDW EM
SSB 0
LB 0.30 Hz
GB 0
PC 1.00

Engy Alber_H_2-1

Microanalytical Unit - FOPCU - NMR laboratory
www.pharma.cu.edu.eg dir-mau.fopcu@pharma.cu.edu.eg

BRUKER

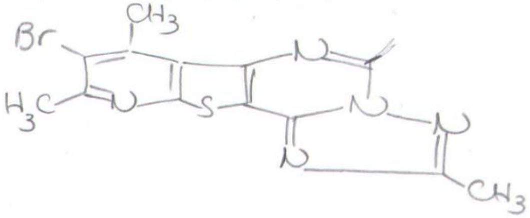


```
Current Data Parameters
NAME      Engy Alber_H_2-1
EXPNO                   10
PROCNO                   1

F2 - Acquisition Parameters
Date_             20170221
Time                 19.19
INSTRUM              spect
PROBHD   5 mm PABBO BB/
PULPROG               zg30
TD                   65536
SOLVENT               DMSO
NS                      32
DS                       2
SWH             8012.820 Hz
FIDRES          0.122266 Hz
AQ             4.0894465 sec
RG                  169.46
DW                  62.400 usec
DE                    6.50 usec
TE                   298.0 K
D1              1.00000000 sec
TD0                      1

======== CHANNEL f1 ========
SFO1         400.1924713 MHz
NUC1                    1H
P1                   15.00 usec
PLW1          10.39999962 W

F2 - Processing parameters
SI                   65536
SF           400.1900000 MHz
WDW                     EM
SSB       0
LB                    0.30 Hz
GB        0
PC                    1.00
```

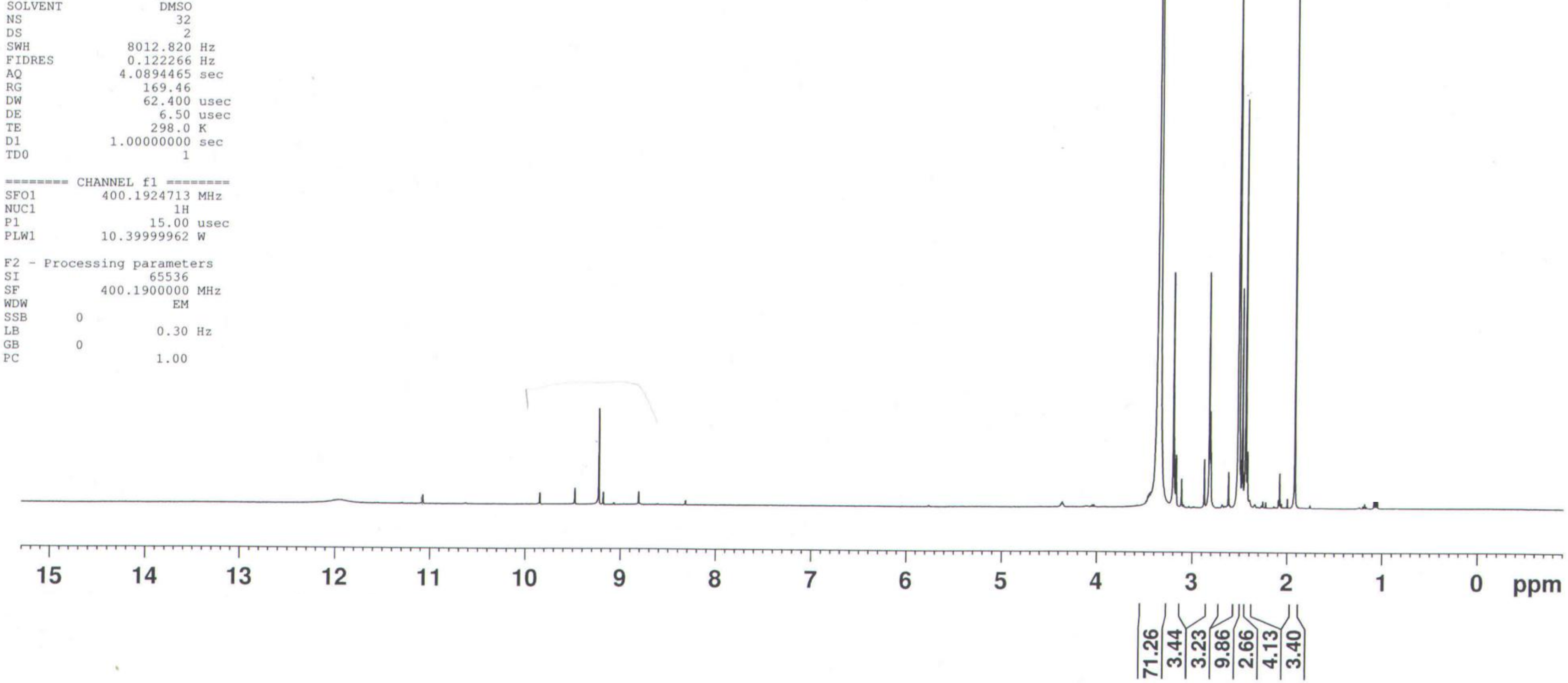

Engy Alber_H_2-3

Microanalytical Unit - FOPCU - NMR laboratory
www.pharma.cu.edu.eg dir-mau.fopcu@pharma.cu.edu.eg

BRUKER

Current Data Parameters
NAME Engy Alber_H_2-3
EXPNO 10
PROCNO 1

F2 - Acquisition Parameters
Date_ 20170322
Time 7.33
INSTRUM spect
PROBHD 5 mm PABBO BB/
PULPROG zg30
TD 65536
SOLVENT DMSO
NS 32
DS 2
SWH 8012.820 Hz
FIDRES 0.122266 Hz
AQ 4.0894465 sec
RG 146.06
DW 62.400 usec
DE 6.50 usec
TE 298.1 K
D1 1.00000000 sec
TD0 1

======== CHANNEL f1 ========
SFO1 400.1924713 MHz
NUC1 1H
P1 15.00 usec
PLW1 10.39999962 W

F2 - Processing parameters
SI 65536
SF 400.1900000 MHz
WDW EM
SSB 0
LB 0.30 Hz
GB 0
PC 1.00

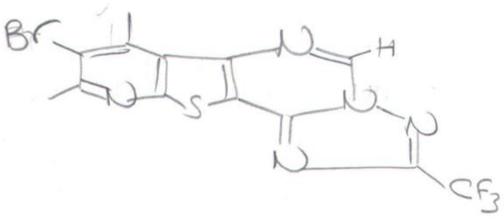

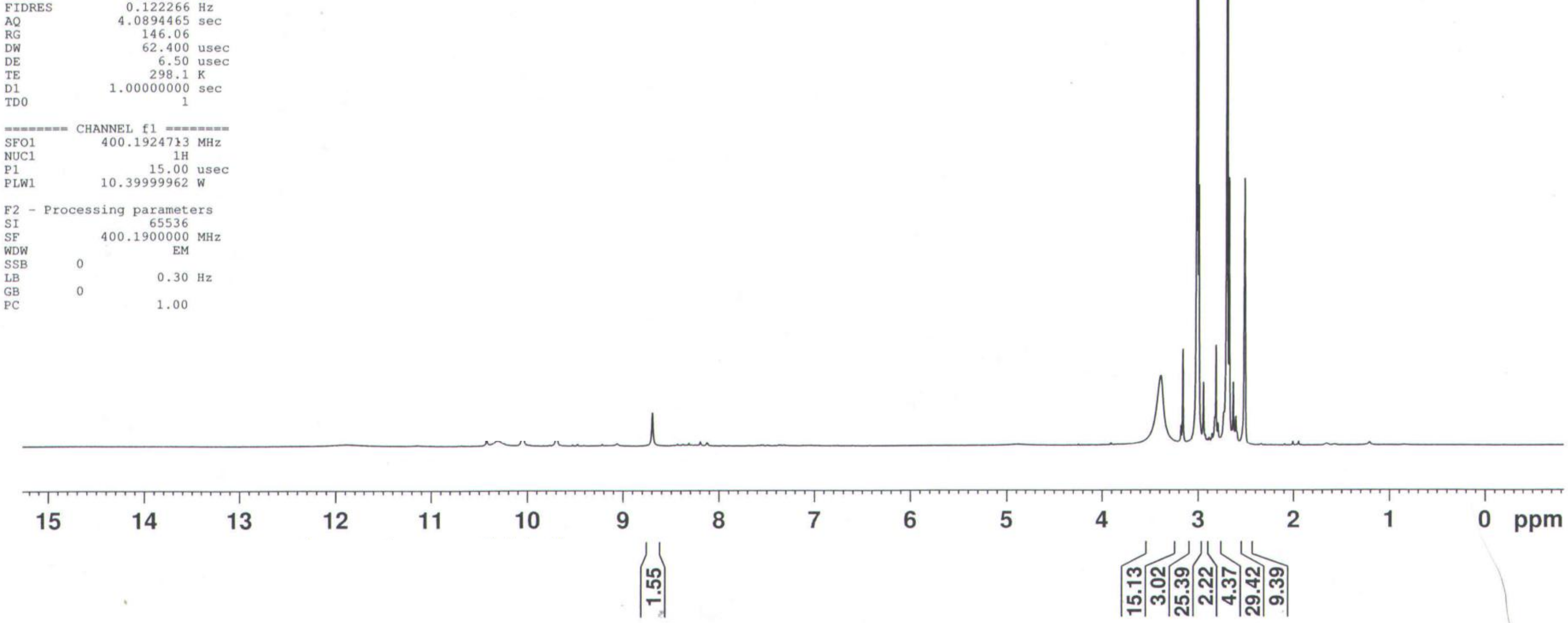

Engy Alber_H_2-8

Microanalytical Unit - FOPCU - NMR laboratory
www.pharma.cu.edu.eg   dir-mau.fopcu@pharma.cu.edu.eg

BRUKER

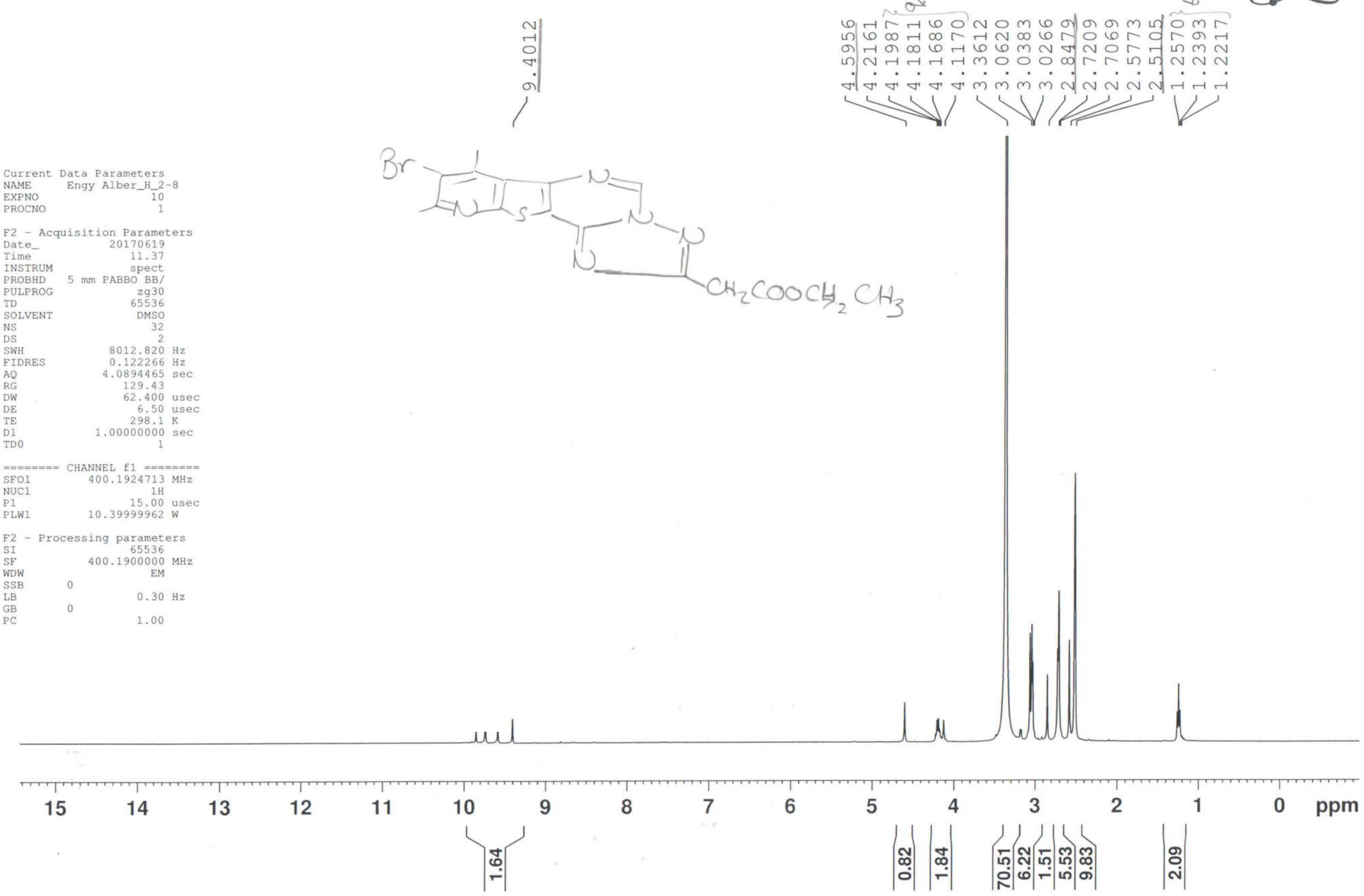


```
Current Data Parameters
NAME      Engy Alber_H_2-8
EXPNO                   10
PROCNO                   1

F2 - Acquisition Parameters
Date_             20170619
Time                 11.37
INSTRUM              spect
PROBHD   5 mm PABBO BB/
PULPROG               zg30
TD                   65536
SOLVENT               DMSO
NS                      32
DS                       2
SWH             8012.820 Hz
FIDRES          0.122266 Hz
AQ             4.0894465 sec
RG                  129.43
DW                62.400 usec
DE                  6.50 usec
TE                 298.1 K
D1           1.00000000 sec
TD0                      1

======== CHANNEL f1 ========
SFO1         400.1924713 MHz
NUC1                    1H
P1                 15.00 usec
PLW1         10.39999962 W

F2 - Processing parameters
SI                   65536
SF           400.1900000 MHz
WDW                     EM
SSB       0
LB                  0.30 Hz
GB        0
PC                   1.00
```

Engy Alber_H_2-7-

Microanalytical Unit - FOPCU - NMR laboratory
www.pharma.cu.edu.eg dir-mau.fopcu@pharma.cu.edu.eg

BRUKER

```
Current Data Parameters
NAME        Engy Alber_H_2-7
EXPNO                     10
PROCNO                     1

F2 - Acquisition Parameters
Date_               20170620
Time                   12.57
INSTRUM                spect
PROBHD     5 mm PABBO BB/
PULPROG                 zg30
TD                     65536
SOLVENT                 DMSO
NS                        32
DS                         2
SWH             8012.820 Hz
FIDRES          0.122266 Hz
AQ             4.0894465 sec
RG                     91.58
DW                62.400 usec
DE                  6.50 usec
TE                   298.0 K
D1            1.00000000 sec
TD0                        1

======== CHANNEL f1 ========
SFO1         400.1924713 MHz
NUC1                      1H
P1                 15.00 usec
PLW1         10.39999962 W

F2 - Processing parameters
SI                     65536
SF           400.1900000 MHz
WDW                       EM
SSB        0
LB                   0.30 Hz
GB         0
PC                      1.00
```

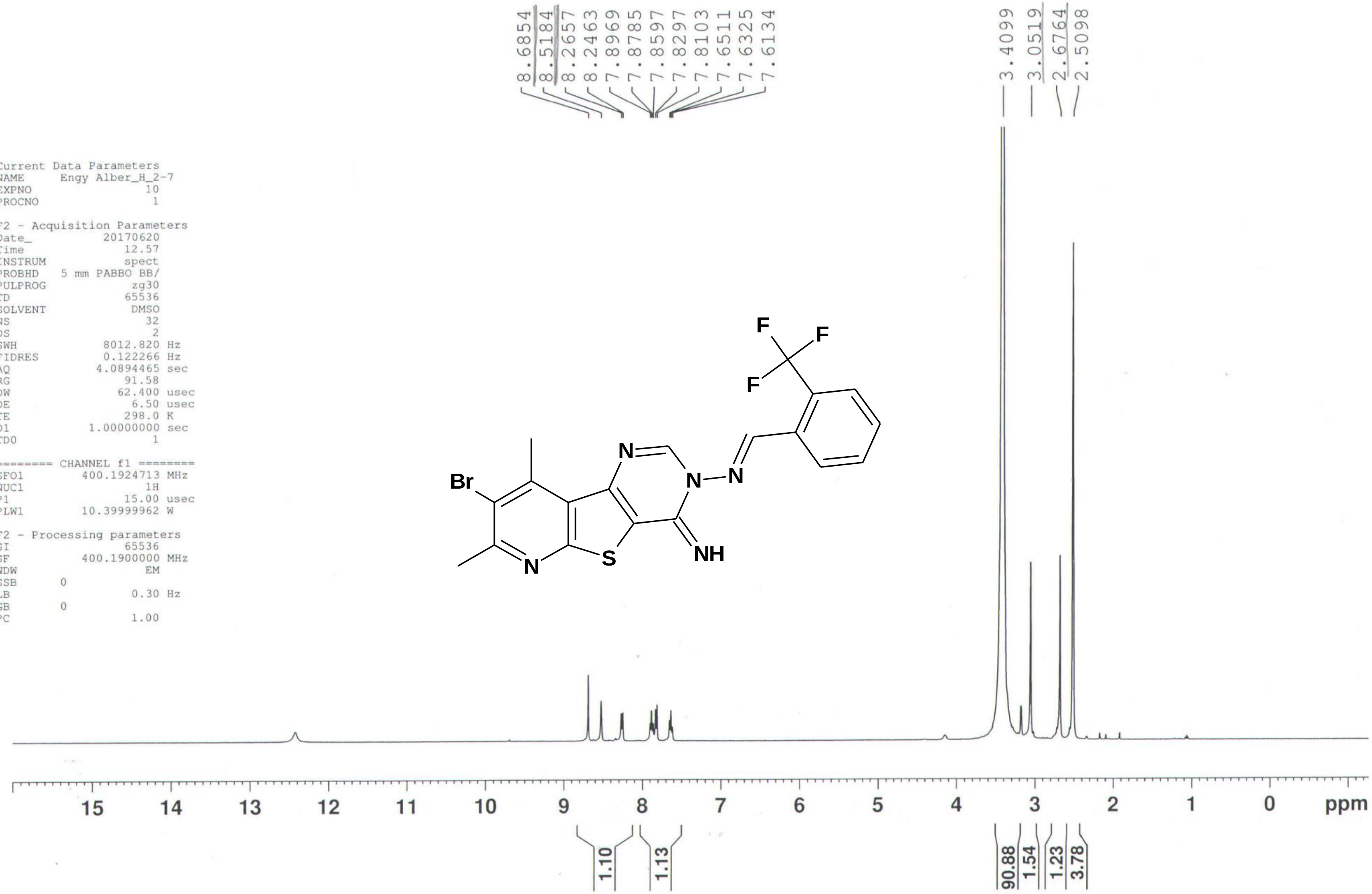

Supplement: IENZ_1389921_Supplementary_Materials.pdf [file IENZ_A_1389921_SM0525.pdf]
